# Computational 3D Modeling-Based Identification of Inhibitors Targeting Cysteine Covalent Bond Catalysts for JAK3 and CYP3A4 Enzymes in the Treatment of Rheumatoid Arthritis

**DOI:** 10.3390/molecules29010023

**Published:** 2023-12-19

**Authors:** Abdelmoujoud Faris, Radwan Alnajjar, Jingjing Guo, Mohammed H. AL Mughram, Adnane Aouidate, Mufarreh Asmari, Menana Elhallaoui

**Affiliations:** 1 LIMAS, Department of Chemical Sciences, Faculty of Sciences Dhar El Mahraz, Sidi Mohamed Ben Abdellah University, Fez 30000, Morocco; melhallaoui@yahoo.fr; 2 Department of Chemistry, Faculty of Science, University of Benghazi, Benghazi 16063, Libya; radwan.alnajjar@uob.edu.ly; 3 PharmD, Faculty of Pharmacy, Libyan International Medical University, Benghazi 16063, Libya; 4 Department of Chemistry, University of Cape Town, Rondebosch 7701, South Africa; 5 Centre in Artificial Intelligence-Driven Drug Discovery, Faculty of Applied Sciences, Macao Polytechnic University, Macao 999078, China; jguo@mpu.edu.mo; 6 Department of Pharmaceutical Chemistry, College of Pharmacy, King Khalid University, Abha 61421, Saudi Arabia; malmogrm@kku.edu.sa (M.H.A.M.); masmri@kku.edu.sa (M.A.); 7 Laboratory of Organic Chemistry and Physical Chemistry, Team of Molecular Modeling, Materials and Environment, Faculty of Sciences, University Ibn Zohr, Agadir 80060, Morocco; a.aouidate@uiz.ac.ma

**Keywords:** drug discovery, 3D-QSAR, structure-based design, small molecule inhibitors, computational chemistry

## Abstract

This work aimed to find new inhibitors of the CYP3A4 and JAK3 enzymes, which are significant players in autoimmune diseases such as rheumatoid arthritis. Advanced computer-aided drug design techniques, such as pharmacophore and 3D-QSAR modeling, were used. Two strong 3D-QSAR models were created, and their predictive power was validated by the strong correlation (R^2^ values > 80%) between the predicted and experimental activity. With an ROC value of 0.9, a pharmacophore model grounded in the DHRRR hypothesis likewise demonstrated strong predictive ability. Eight possible inhibitors were found, and six new inhibitors were designed in silico using these computational models. The pharmacokinetic and safety characteristics of these candidates were thoroughly assessed. The possible interactions between the inhibitors and the target enzymes were made clear via molecular docking. Furthermore, MM/GBSA computations and molecular dynamics simulations offered insightful information about the stability of the binding between inhibitors and CYP3A4 or JAK3. Through the integration of various computational approaches, this study successfully identified potential inhibitor candidates for additional investigation and efficiently screened compounds. The findings contribute to our knowledge of enzyme–inhibitor interactions and may help us create more effective treatments for autoimmune conditions like rheumatoid arthritis.

## 1. Introduction

Rheumatoid arthritis (RA) is a chronic inflammatory disease that significantly impacts the immune system [1]. It is characterized by persistent inflammation of the synovial joint lining, leading to substantial financial burdens, premature mortality, and a progressive decline in quality of life [2,3] This condition predominantly affects women and tends to manifest in older individuals [4]. Early identification of RA within the onset of symptoms is crucial for achieving optimal therapeutic outcomes, including limiting joint damage, preserving joint function, and minimizing radiological progression [5]. RA is the result of a combination of genetic and environmental factors, including variants of the major histocompatibility complex class II, tumor necrosis factor associated with factor 1 receptor, exposure to silica dust, and smoking, among others [6]. The underlying mechanisms of this disease remain partially understood, with autoimmune responses known to develop several years before the clinical symptoms become apparent [7].

Among the relevant molecular targets, Janus kinases (JAKs) are promising targets due to their essential role in regulating the JAK signal transducer and activator of the transcription (STAT) signaling pathway, which is associated with inflammation and immunity [8]. JAK inhibitors, including JAK1, JAK2, JAK3, and TYK2, have emerged as potential treatments for RA, with some of them receiving approval for use in this disease [9]. These medications modulate JAK signaling, reducing inflammation and alleviating symptoms while preserving joint integrity in RA patients [10]. Understanding the interactions between JAK inhibitors, with JAK3, and CYP3A4 is essential for developing safer and more effective treatments for RA [11]. Docking molecular studies have provided crucial preliminary information, but experimental validation remains imperative to ensure the efficacy and safety of JAK inhibitor-based treatments [12,13]. JAK enzymes are integral to transmitting signals from various cytokines and growth factors [14]. Recently, attention has focused on JAK3 (Janus kinase 3) as an important target in the treatment of RA, given its specific role in the cytokine signaling pathway [15]. In the context of RA, cytokine signaling plays a central role in chronic inflammation and joint damage, making JAK3 a preferred target [16]. It is noteworthy that some JAK inhibitors target both JAK3 and JAK1, but selectively targeting JAK3 offers potential advantages in terms of specificity and the profile of adverse effects [17]. It is essential to emphasize that decisions regarding the use of JAK-targeting medications for RA treatment should be made by qualified healthcare professionals, considering each patient’s individual needs and the associated risks and benefits of these drugs [10,18].

The metabolism of JAK inhibitors can also be influenced by other enzymes, including cytochrome P450 3A4 (CYP3A4), which is a cytochrome P450 enzyme found in the liver [19]. This enzyme plays a crucial role in the metabolism of many drugs, including some JAK inhibitors [20,21]. Inhibition of CYP3A4 can impact the efficacy and safety of treatment by altering the plasma concentrations of drugs metabolized by this enzyme [22]. Tofacitinib is a selective inhibitor of JAK3 that is used to treat certain autoimmune diseases, including rheumatoid arthritis, which helps reduce the activity of the immune system [15]. Tofacitinib is primarily metabolized by the enzyme CYP3A4 [23], and the inhibition of CYP3A4 can impact the tofacitinib metabolism, potentially leading to drug interactions [12,20,21]. Inhibition of CYP3A4 can potentially affect the metabolism and efficacy of tofacitinib, highlighting the importance of considering drug interactions when using it [24,25,26].

Herein, in this study, a series characterized by the presence of 1,4 Michael groups was investigated. The QSAR model allows for the design of molecules with groups that favor the Michael 1,4 reaction, while the DHRRR pharmacophore model is used to identify groups associated with α,β-unsaturated ketones through screening a database from PubChem, including JAK3 inhibitors. The α,β-unsaturated ketone is a functional group found in organic molecules, characterized by the presence of a carbonyl group (C=O) next to a double bond. The α,β-unsaturated ketones can undergo various chemical reactions, including the Michael 1,4 addition. This reaction involves the nucleophilic addition of a nucleophile to an acceptor enone, forming a bond between the β-carbon of the enone and the nucleophile. Table 1 provides a summary of the advantages and limitations of each reaction. It highlights the positive aspects, such as wide applicability and structural diversity in the case of covalent bond formation with a α,β-unsaturated ketone, as well as the selective formation of C-C bonds and simple reaction steps in the case of the Michael 1,4 reaction [27,28]. However, it also emphasizes limitations such as the limited reactivity of certain substrates and competitive reactions in the case of the Michael 1,4 reaction, as well as the formation of undesired by-products in the case of covalent bond formation with an α,β-unsaturated ketone [29].

Today, the significance of computer-aided drug design (CADD) in chemistry for the discovery of new medications is undeniable [30,31,32,33]. This approach plays a pivotal role in identifying promising pharmaceutical compounds for the treatment of various diseases, including rheumatoid arthritis (RA). In this study, diverse CADD methodologies have facilitated the design and identification of a series of molecules exhibiting potent experimental inhibitory activity against JAK3, positioning them as potential candidates for potent RA treatments [34,35]. Three-dimensional models have guided the design and identification of these novel JAK3 and CYP3A4 inhibitory molecules, while both field-based and atom-based quantitative structure–activity relationship (QSAR) approaches, as well as pharmacophore modeling, have yielded significant results, with favorable validation. Furthermore, molecular dynamics (MD) simulations and MM/GBSA solvation energy have corroborated the findings from molecular docking, demonstrating that these newly designed molecules can form irreversible covalent bonds with Cys909 and reversible bonds with CYP3A4. These new molecules can thus be regarded as promising targets for the development of novel medications aimed at treating rheumatoid arthritis.

## 2. Results and Discussion

### 2.1. Static Results of QSAR Models

#### Static Analysis of Field-Based and Atom-Based Models

The field-based QSAR model was generated based on a training set of 59 molecules used to build the model (Table 2). As shown in Table 2, the best model (PLS factor = 4) offered good predictive power. The Leave-One-Out (LOO) method of cross-validation was adopted for the assessment of the predictive abilities of the models. The cross-validated value R^2^_CV_ was 0.53. The R^2^ for the regression was 0.80, and the stability value was 0.80. The P value (6.13 × 10^−15^) also suggested a greater degree of confidence. The reliability of the model was tested via an external test set of 16 compounds. The RMSE (0.44), Q^2^ (0.763), and Pearson-r (0.88) models all further confirmed their robustness (Table 2). Activity residual values for the training set and the test set are shown in Table S1.

**Figure 1:**
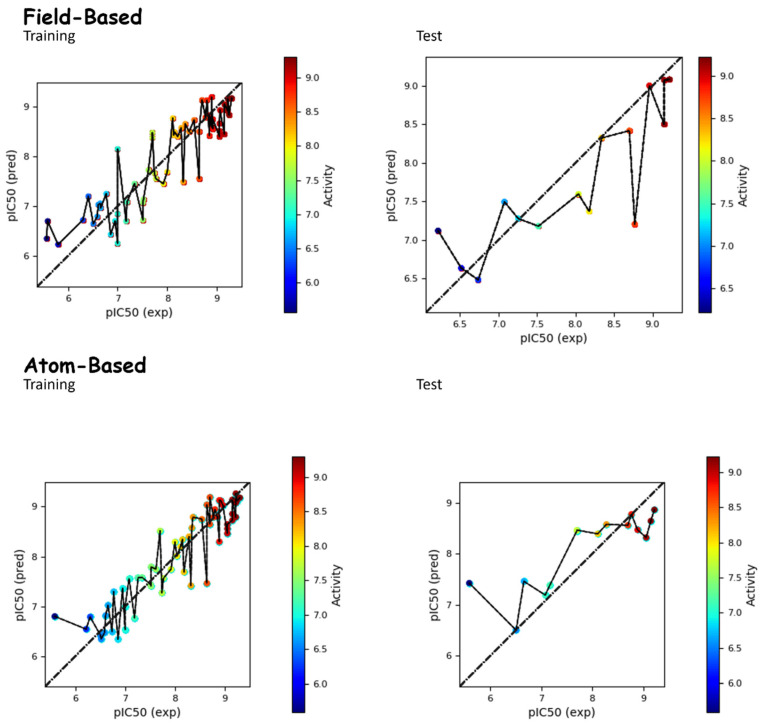
Comparison between the actual and predicted pIC_50_ values of molecules in both the test and training sets for both field-based and atom-based approaches.

The atom-based QSAR model was generated based on a training set of 59 molecules that was used to build the model (Table 3). As shown in Table 3, the best model (PLS factor = 4) offered good predictive power. The Leave-One-Out (LOO) method of cross-validation was adopted for the assessment of the predictive abilities of the models. The cross-validated value R^2^_CV_ was 0.56. The R^2^ for the regression was 0.87, and the stability value was 0.60. The *p* value (2.91 × 10^−20^) also suggested a greater degree of confidence. The reliability of the model was tested via an external test set of 13 compounds. The RMSE (0.66), Q^2^ (0.66), and Pearson-r (0.82) of the model all further confirmed its robustness (Table 3). The correlation between the experimental and predicted activities for the dataset is displayed in Figure 1.

### 2.2. Contour Maps

**Figure 2:**
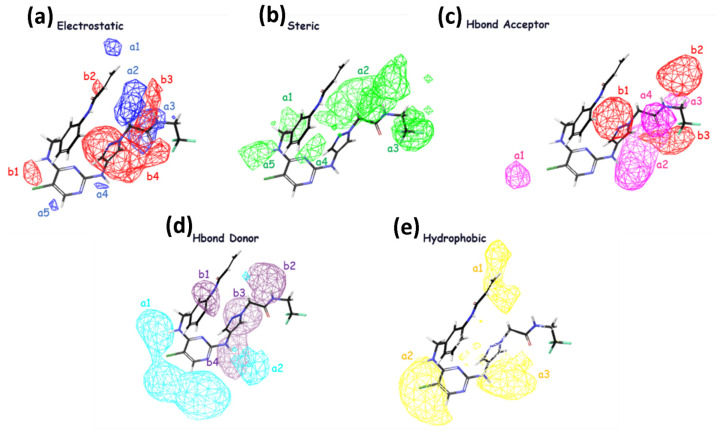
The contour maps generated for the test set compounds depict different fields. Specifically, (**a**) Gaussian electrostatic fields are represented by blue color for favored electropositive regions and red color for disfavored electronegative regions. (**b**) Gaussian hydrogen bond acceptor fields are depicted using red color for favored regions and magenta color for disfavored regions. (**c**) Gaussian hydrogen bond donor fields are shown in purple for favored regions and cyan for disfavored regions. (**d**) Gaussian steric fields are displayed in green for favored regions and yellow for unfavorable regions. (**e**) Gaussian hydrophobic fields are represented by yellow color for favored regions and white color for disfavored regions.

The significance of steric, hydrophobic, electrostatic, and hydrogen bond interactions (both as acceptor and donor) in explaining the studied properties is emphasized in field-based Model 4. This finding is consistent with the order of importance for increasing inhibitory activity, indicated by the coefficients reported in Table 4. The coefficients reveal that the steric group has the highest value, followed by hydrophobic, hydrogen bond acceptor, hydrogen bond donor, and electrostatic interactions, respectively, as presented in Table 4. In Figure 2, it is observed that the inhibitory activity is enhanced with groups that can generate electrostatic effects, favoring regions b3 and b4 with larger contours, suggesting their importance in these regions. The blue cubes represent regions where activity is favored, while the red regions correspond to disfavored regions for activity enhancement in regions a2 and a3. Regarding steric effects, it is noted that in regions a1–a5, increased biological activity is favored in the presence of more substituted groups. H-bond acceptor groups, depicted by larger contours in b1–b3, suggest the importance of H-bond donor groups in these regions, while the pink groups in a1–a4 disfavor the enhancement of inhibitory activity in these regions. For H-bond donor groups, all b1 groups in purple favor an increase in inhibitory activity, whereas the groups in cyan in regions a1–a2 disfavor the enhancement of inhibitory activity in the presence of H-bonds. In terms of hydrophobicity, the yellow contours suggest that in regions a1–a3, substituted hydrophobic groups favor an increase in inhibitory activity.

**Figure 3:**
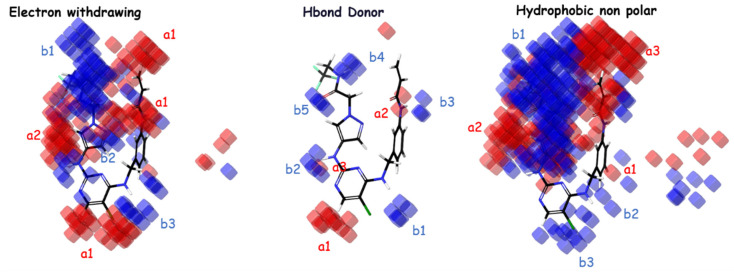
The atom-based PHASE 3D-QSAR model utilizes visual representations to depict different molecular characteristics, such as electron-withdrawing, hydrogen bond donor, and hydrophobic properties. In these representations, blue-colored cubes indicate positive coefficients or an increase in activity, while red-colored cubes indicate negative coefficients or a decrease in activity.

The importance of hydrophobic/nonpolar and electron-withdrawing interactions in the atom-based Model 4 is highlighted by these factors. According to the important coefficients presented in Table 4, the groups that most favor an increase in inhibitory activity, in descending order, are hydrophobic/nonpolar, electron-withdrawing, and hydrogen bond donor. The analysis of Figure 3 for electron-withdrawing in the indicated regions shows a distribution with similarities of blue/red cubes, suggesting that the corresponding electron-withdrawing groups correspond to favorable/unfavorable inhibition activity. Regarding H-bond donors, it can be observed from the distribution of blue/red cubes that in regions a1, b1, b2, and b4, the presence of groups with H-bonds favor/disfavor an increase in inhibitory activity. For hydrophobic nonpolar groups, it is evident that regions b2 and b3 favor an increase in inhibitory activity in the presence of hydrophobic groups compared to other regions, which exhibit a similar distribution of blue/red cubes.

### 2.3. Design of New Molecules Based on QSAR Models

**Figure 4:**
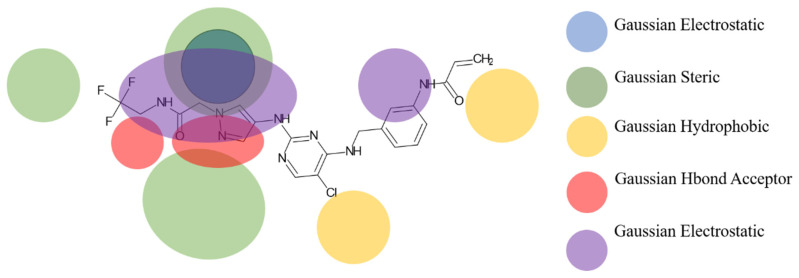
Regions favoring the development of inhibitory activity pIC_50_.

Based on the best models obtained from field-based and atom-based, the scheme in Figure 4 facilitates the analysis for designing new molecules presented in Table 5.

### 2.4. Pharmacophore Model Analysis

**Figure 5:**
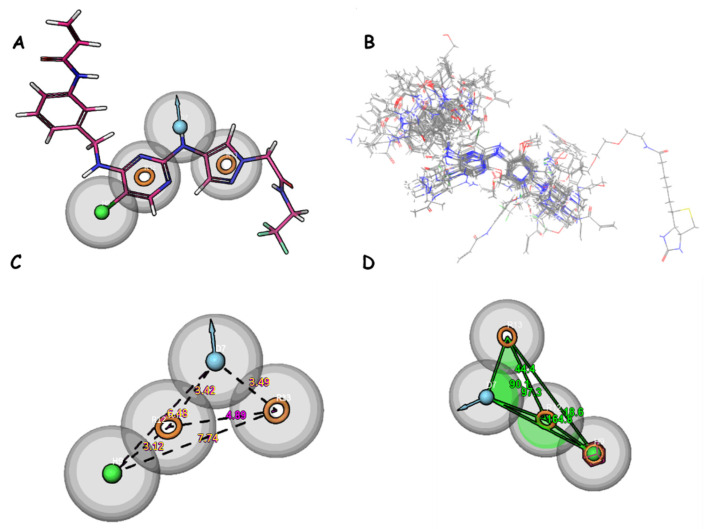
(**A**) Pharmacophore features of DHRRR_1; (**B**) alignment of studied compounds; (**C**) distance between features; and (**D**) angles between features. Yellow circles represent aromatic rings. The blue balls indicate donor groups, while the green balls indicate hydrophobic groups.

The results of the pharmacophore hypothesis analysis include different scores for several models. These scores are utilized to assess the quality of pharmacophore models and their relevance for the identification of active chemical compounds. In this section, the results for the DDHRR_1 model will be discussed, along with global scores for other models for comparison (Figure 5). 

For the DDHRR_1 model, a survival score of 6.265 is achieved, a site score of 0.837 is attained, a vector score of 0.951 is recorded, a volume score of 0.765 is registered, a BEDROC score of 0.932 is obtained, and a PhaseHypoScore of 1.308 is measured (Table 6 and Table 7). The survival score results from a combination of these scores with adjustable weights determined by the user. Scoring is executed on ligands within the active set, constituting ligands utilized in hypothesis development, for all selected variants listed in the variants table within the find common pharmacophores step. The vector score represents the average of the cosines of the angles between the vectors of each aligned ligand and those of the reference ligand. A good survival score is achieved by this model, indicating that active compounds can be effectively identified by it. A notably high site score is also achieved, suggesting precision in locating active sites. Furthermore, a high BEDROC score is recorded, indicating that active compounds can be rapidly retrieved by this model. Among models with comparable survival scores, DDHRR_1 stands out due to its high site score, implying that active site prediction is conducted exceptionally well by it. Additionally, when compared to similar models, DDHRR_1 boasts a high vector score, indicating that better discrimination between different spatial orientations of molecules is achieved. In terms of volume and BEDROC, DDHRR_1 is also competitive when compared to other models.

#### Validation of Pharmacophore Models

It is essential to consider several metrics to evaluate the performance of a binary classification model. ROC (alpha) and RI (alpha) provide an overall assessment of the model’s discriminatory ability, but it is also important to consider other metrics such as precision, recall, and F-measure. These metrics allow us to assess the model’s performance at different classification thresholds and account for the imbalance between positive and negative classes. The results indicate that the pharmacophore model DHRR_1 achieved good performance scores, with an EF1% of 1.31. BEDROCK (alpha) and RI (alpha) are performance evaluation metrics for a binary classification model. ROC (alpha) is the area under the ROC curve using the alpha threshold for classification, and RI (alpha) is the true positive rate at a false positive rate corresponding to the alpha threshold (Table 7). These metrics are often used to evaluate a model’s ability to discriminate between positive and negative classes. It suggests that the ranking and scoring method used in the DHRR_1 analysis successfully captured all the known activities in the ranked list. 

Among the 75 evaluated ligands, including both actives and decoys, a total of 58 molecules are identified as actives. This means that all known active molecules are included in the ranked list of ligands. These results suggest that the ranking method used for the DHRR_1 analysis effectively identified all known active molecules in the ranked list. The provided results suggest that the hypothesis phase achieved a high hypothesis PhaseHypoScore, strong enrichment (EF1%) in the top 1% of the results, and perfect matches for all expected targets. The high BEDROC160.9 score further indicates good early enrichment performance. The percentage of actives increases as we consider a larger percentage of decoys. In the top 10% of decoys, there are five actives, representing 10.4% of the total actives. The top 20% of decoys have 19 actives, representing 39.6% of the total actives. As we consider a larger percentage of results, the count and percentage of actives increase. In the top 2% of results, one active represents 2.1% of the total actives. In the top 5% of results, three actives represent 6.2% of the total actives. In the top 10% of results, five actives represent 10.4% of the total actives. In the top 20% of results, 11 actives represent 22.9% of the total actives. The enrichment factors (EFs) indicate a modest enrichment of actives in the sampled data, with EF values ranging from 1.1 to 1.3 depending on the sample size or the percentage of actives recovered.

The results demonstrate that the DHRR_1 model achieved strong performance in capturing known active molecules. The hypothesis phase showed high scores and strong enrichment, indicating its effectiveness in identifying active compounds. Additionally, the analysis revealed an increasing percentage of actives as larger result percentages were considered, suggesting a promising enrichment trend. These findings highlight the model’s ability to discriminate between positive and negative classes and its potential for identifying active compounds.

**Figure 6:**
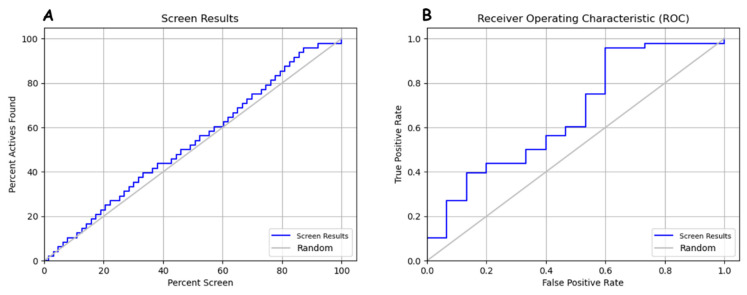
(**A**) Relationship between percent active found and percent screen in model evaluation. (**B**) ROC curve analysis for pharmacophore model.

In Figure 6, it appears that the screen results curve behaves in a stair-step fashion in the true positive rate (TPR) zone, remaining above the random curve on an ROC graph. The fact that the screen results curve stays above the random curve in the TPR zone indicates that model DHRRR_1 performs better than mere random chance for detecting true positives. In other words, it can reliably detect positive examples. This configuration suggests that the pharmacophore or screening model strongly detects true positives while maintaining a relatively low false positive rate. This can be a good indicator of the ability to discriminate between positive and negative. Interpreting the relationship between the percentage of active compounds found (percent active found) and the percentage of screening (percent screen) about the screen results and random curves will help evaluate the pharmacophore model’s ability to identify actives in each screening set. Better performance is indicated by the screen results curve being above random and a rapid increase in the percentage of active compounds found as the percentage of screening increases.

### 2.5. Identification of Compounds Using Pharmacophore Model DHRRR_1

The newly identified molecules obtained through the utilization of the DHRRR_1 pharmacophore model by screening a database of JAK3 inhibitor structures obtained from the literature and PubChem comprise a total of 568 compounds. The new compounds are identified based on the pharmacophore model in Figure 5, provided that the molecules exhibit a low RMSD of less than 0.5 Å and the structures of the compounds show a comparable similarity with the identified characteristic groups in the DHRR pharmacophore module (Table 8).

### 2.6. ADMET Analysis

According to ADMET rules [36,37], for designing new compounds (Table 9 and Table 10), the value of logS reflects the drug’s solubility. The smaller the value, the less soluble the compound is in water. When logS are less than −6.0, the compounds are considered poorly soluble and insoluble. A molecule with less than 30% absorption is considered weakly absorbed, while molecules with an absorption greater than 30% are considered to have high absorption. The unit of BBB penetration is cm/s. Molecules with logBB greater than −1 are classified as BBB^+^ (Category 1), while molecules with logBB less than or equal to −1 are classified as BBB^−^ (Category 0). BBB^−^ indicates that the molecule has a low capacity to penetrate the blood–brain barrier (BBB) or does not penetrate at all. This may be desirable for certain drugs targeting the central nervous system (CNS) to minimize side effects or undesirable interactions with the brain. BBB^+^ indicates that the molecule has a high capacity to penetrate the BBB. This may be desirable for certain drugs that require direct access to the brain to be effective in treating CNS diseases. The output value represents the probability of being BBB^+^, ranging from 0 to 1. Molecules **D1**, **D2**, **D3**, and **D6** did not show BBB penetration, whereas **D4** and **D5** demonstrated BBB penetration, suggesting a risk to the CNS. Since the inhibition of CYP3A4 remains a therapeutic target for diseases, especially rheumatoid arthritis, the results suggest that compounds **D1**, **D2**, and **D5** could inhibit CYP3A4. The total clearance constant (TC), which indicates the drug’s clearance, is used to evaluate the drug’s half-life time. A low TC value indicates a long half-life time of the drug. It encompasses both hepatic and renal clearance and is important for bioavailability and determining dosage rates to achieve steady-state concentrations. All compounds exhibit low total clearance, ranging from −0.059 to 0.19 mL/min/kg, indicating a long half-life. For toxicity, the molecules did not show a positive AME test result.

**Figure 7:**
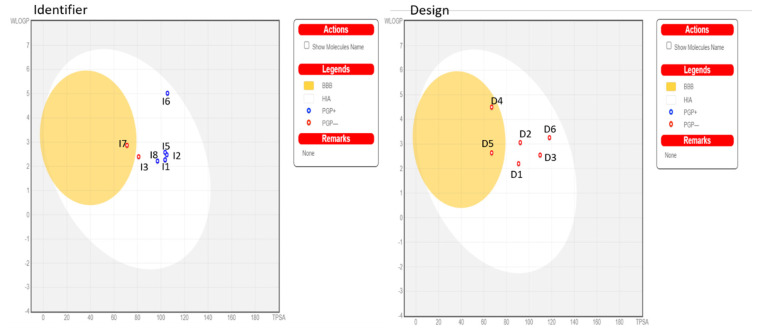
SwissADME analysis of boiled egg.

Similarly, in the ADMET analysis of the identified compounds, all molecules with logS greater than −6 suggest solubility. For absorption, all molecules exhibit high absorption rates above 30%. Regarding penetration, all molecules, except I7, penetrate the BBB and are classified as BBB^−^. In contrast, the other molecules are not classified as BBB^+^ and do not penetrate the BBB (Figure 7). For the inhibition of CYP3A4, molecules **I1**, **I4**, **I5**, and **I6** demonstrate CYP3A4 inhibition. Regarding total clearance, the molecules show low clearance values ranging from 0.48 to 0.70 mL/min/kg. As for toxicity, molecules **I1**, **I2**, **I4**, **I5**, **I7**, and **I8** do not yield a positive AMES test result compared to the other molecules. **D1**, **D2**, and **I1** have been identified as the top compounds for the MD based on the ADMET rules, as well as their strong affinity.

### 2.7. Analysis Docking of Selected Molecules (Covalent Docking between Identified and Designed Molecules)

**Figure 8:**
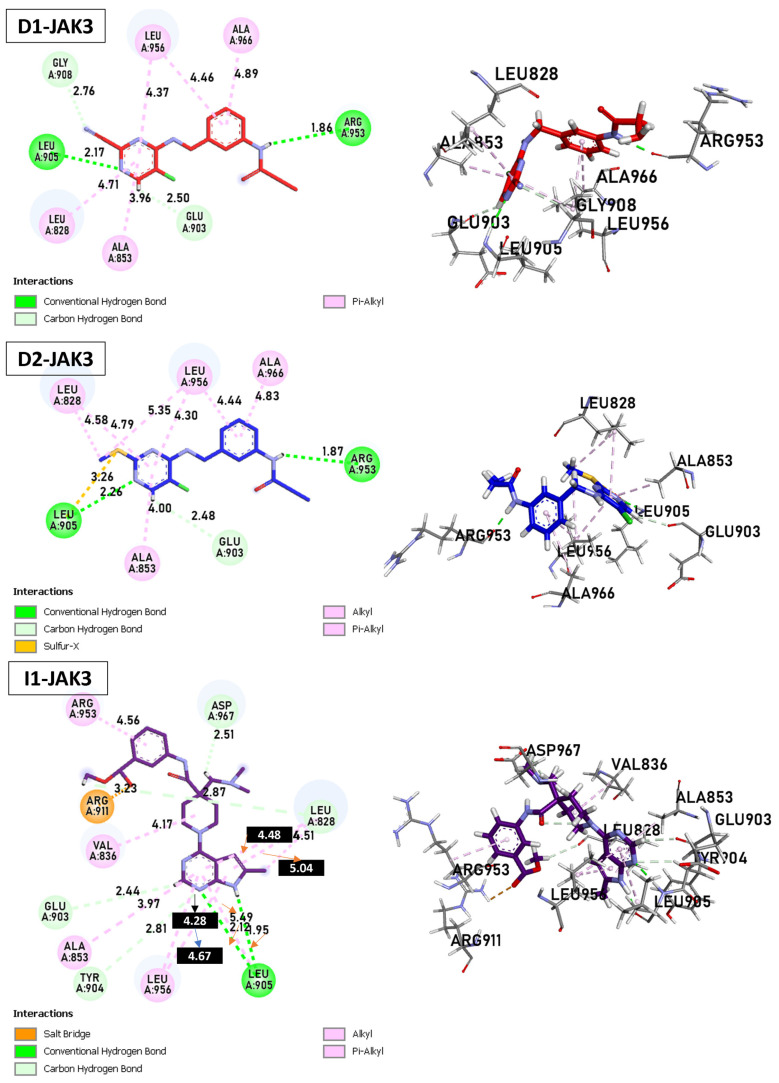
Binding interactions of new compounds **D1**, **D2**, and **I1** with JAK3 (PDB ID: 4Z16), illustrated in 3D and 2D diagrams.

Today, covalent docking has emerged as an irreversible method with great potential for targeting autoimmune diseases, specifically focusing on the protein JAK3 (ID: 4Z16). JAK3 is one of the pioneering proteins that exhibit remarkable resolution and demonstrates a superior fit to experimental data through the better ligand structure. A co-crystallized ligand (4LH) has been identified, which forms a strong covalent bond with Cys909 by employing an acrylaldehyde moiety through a 1,4 Michael addition reaction. In this context, ligands **D1** and **D2** have been carefully selected and designed, incorporating a similar acrylaldehyde moiety (Figure 8). On the other hand, ligand **I1** utilizes an ester group and undergoes covalent docking to establish its binding (Figure 8).

**Figure 9:**
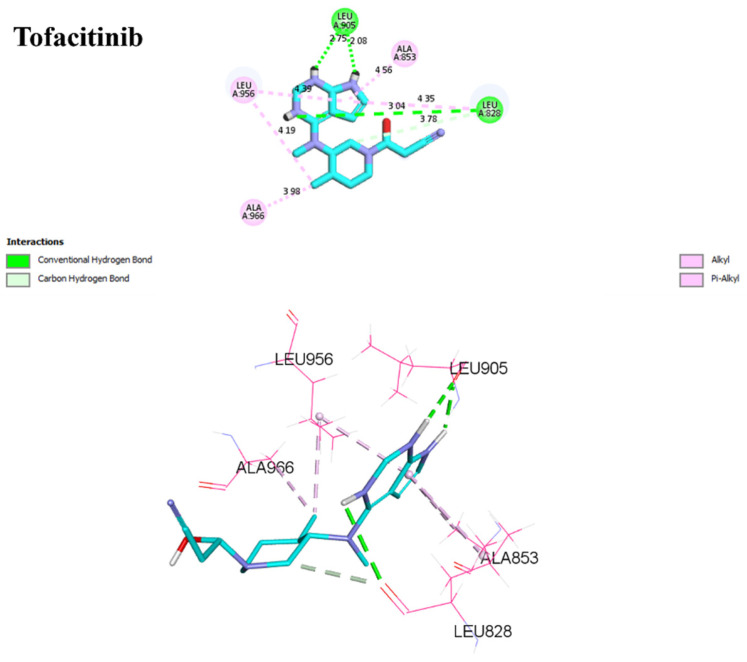
Binding interactions of tofacitinib with JAK3 (PDB ID: 4Z16), illustrated in 3D and 2D diagrams.

For **D1**, residues Arg953 and Leu905 form hydrogen bonds with distances of 1.86 and 2.17 Å. Leu828, Ala853, Leu956, and Ala966 form π–alkyl interactions with distances ranging from 3.96 to 4.89 Å. In the case of **D2**, Arg953, and Leu905 once again form hydrogen bonds, with distances of 1.87 and 2.26 Å, respectively. A sulfur–X interaction is observed towards Leu905, Leu828, and Leu956 at distances of 3.26, 4.58, and 4.79, respectively. The same π–alkyl-type interactions are observed toward residues Leu828, Ala853, Leu956, and Ala966, with similar distances to **D1**. Finally, **I1** formed a salt bridge interaction with Arg911, with 3.23 Å. **I1** forms two hydrogen bonds with Leu905, with respective distances of 2.12 and 1.95 Å. The π–alkyl and VdW interactions are observed between aromatic residues up to 3 Å. In Figure 9, tofacitinib formed multiple hydrogen bonds with Leu828 and Leu906 at distances of 3.04, 2.75, 2.08, and 3.78 Å. Additionally, hydrophobic interactions were observed between tofacitinib and residues Leu956, Ala966, Leu828 (4.35 Å), Ala853 (4.56 Å), and Leu956 (4.39 Å).

Compared to the tofacitinib drug, compounds **D1**, **D2**, and **I1** all form key hydrogen bonding and electrostatic interactions with the target protein, explaining their affinity. In the presence of covalent bonds with Cys909 and hydrogen bonds, non-covalent interactions increase, thereby enhancing affinity and promoting ligand binding.

### 2.8. Molecular Docking with CYP3A4

**Figure 10:**
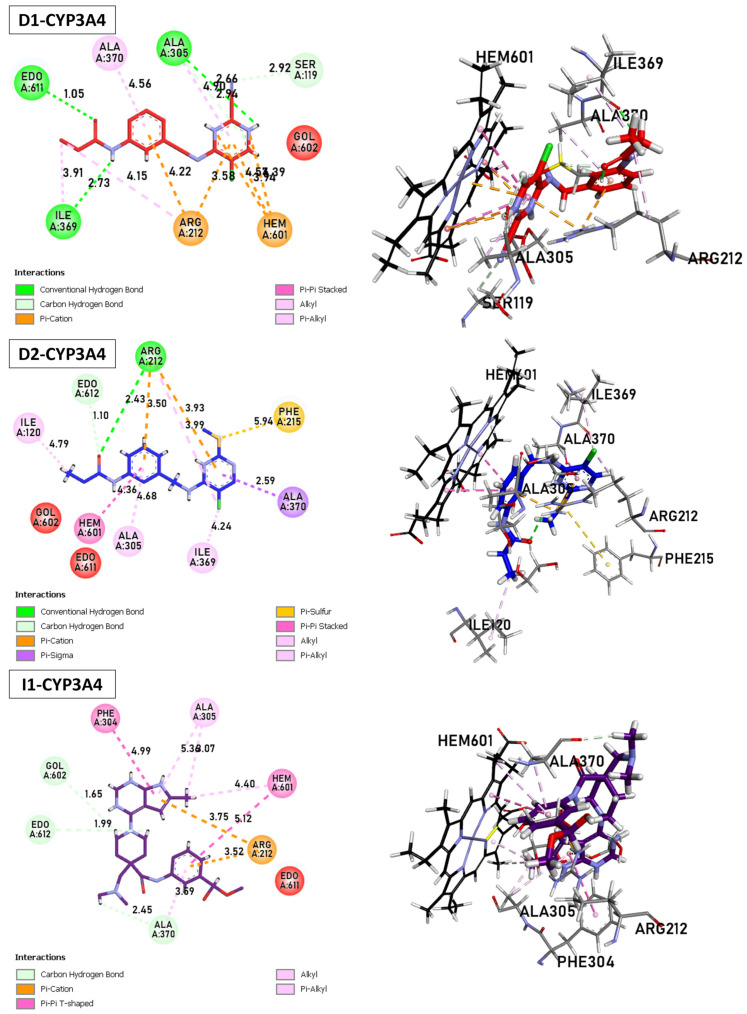
Binding Interactions of new compounds **D1**, **D2**, and **I1** with CYP3A4 (PDB ID: 5VCC), illustrated in 3D and 2D diagrams.

The metabolism of the enzyme CYP3A4, involving compounds **D1**, **D2**, and **I2**, was investigated (Figure 10). The enzyme (ID: 5VCC) selected for this study also exhibits a high resolution and good fit to experimental data through the better ligand structure. This protein choice provides valuable insights into the metabolic processes within the enzyme. Notably, a co-ligand, HEM, remains a target of interest due to its ability to activate CYP3A4. Consequently, there is a focus on inhibiting this enzyme as a potential strategy to modulate its activity.

**D1**: The shortest hydrogen bond is formed between Arg212 and Hem601, with 3.24 Å. Other hydrogen bonds are observed with Ala305 (2.66 Å), Ile369 (2.73 Å), and Ser119 (3.49 Å). Carbon–hydrogen bond interactions are also present with Ala305, Ser119, and Ala370. The π–cation interactions between Arg212 and Hem601/Hem601 are observed, with distances ranging from 3.58 to 4.22 Å. Several stacked π–π and T-shaped interactions between Hem601/Hem601 and aromatic residues of CYP3A4, such as Phe215 and Phe304. Finally, alkyl interactions are present between Hem601 and Ile369, Ile120 and Ala305, as well as between Arg212 and Hem601. **D2**: Similar to **D1**, the shortest hydrogen bond involves Arg212 and Hem601, with 3.14 Å. Another bond of this type is formed with Ile369 (4.24 Å). The π–cation and alkyl interactions are observed between Arg212 and Hem601. Numerous stacked π–π and π–alkyl interactions are found between Hem601 and aromatic residues Phe and Ala. **D3** The shortest carbon–hydrogen bonds are established between Edo612/Gol602 and Hem601, with distances of 1.99 and 1.65 Å, respectively. The π–cation interactions (3.52–3.75 Å), π–H-bond donor (3.42 Å), and T-shaped π–π interactions are present between Arg212, Ser119, Ala370, and Hem601. Finally, alkyl interactions involve the residues Hem601, Ala305, and Ala370.

**D1** and **D2** inhibitors, as well as I1, target the Hem molecule to decrease its activity, which in turn affects the activity of CYP3A4. The shortest hydrogen bond in both **D1** and **D2** inhibitors involves Arg212 and Hem601. This interaction suggests a strong binding between the inhibitor and the target molecule. Additionally, other hydrogen bonds are observed with different residues, such as Ala305, Ile369, and Ser119, indicating multiple binding sites and potential stability of the inhibitor Hem complex. Both **D1** and **D2** inhibitors exhibit π–cation interactions between Arg212 and Hem601, indicating the presence of positive charges in the inhibitor that interact with the π–electron system of Hem601. These interactions contribute to the binding affinity between the inhibitor and Hem.

Furthermore, alkyl interactions are observed between the inhibitor and various residues, suggesting hydrophobic interactions that can enhance the stability of the inhibitor–Hem complex. **D1** specifically shows stacked π–π and T-shaped interactions between Hem601 and aromatic residues of CYP3A4, such as Phe215 and Phe304. These interactions indicate potential binding between the inhibitor and the aromatic residues of the target enzyme, which can affect the enzyme’s activity by interfering with its active site or substrate binding. In **D2**, numerous stacked π–π and π–alkyl interactions are found between Hem601 and aromatic residues Phe and Ala. These interactions suggest a strong binding between the inhibitor and the target molecule, potentially disrupting the activity of CYP3A4. Overall, these inhibitors (**D1**, **D2**, and **I1**) exhibit various interactions with the Hem molecule, including hydrogen bonds, π–cation interactions, and alkyl interactions. These interactions contribute to the inhibition of Hem activity and subsequently impact the activity of CYP3A4, a crucial enzyme in drug metabolism.

### 2.9. Molecular Dynamics Simulation Analysis

#### 2.9.1. RMSD, RMSF, RoG, and SASA Analyses

The analyses of RMSD, RMSF, RoG, SASA, flexibility, and PCA provide crucial insights into different aspects of the studied complexes. RMSD measures the average deviations of atomic positions between initial and final structures, enabling the assessment of stability and conformational variations in the complexes. RMSF quantifies the average fluctuations of atomic positions during the simulation, revealing the flexibility of residues and their relative stability. RoG measures the three-dimensional compactness of a molecule, providing information about the size and shape of the studied complex. SASA evaluates the solvent-accessible surface of a molecule, giving insights into the accessibility of residues and their exposure to the environment. FEL measures a molecule’s ability to deform or change conformation, thus revealing its structural plasticity. PCA analysis is a statistical technique that reduces data complexity by identifying the main modes of variation in the studied structures. These parameters yield results that enable the assessment of stability, flexibility, compactness, solvent accessibility, interactions, and variation modes of the studied complexes, contributing to a better understanding of their structural behavior and properties.

**Figure 11:**
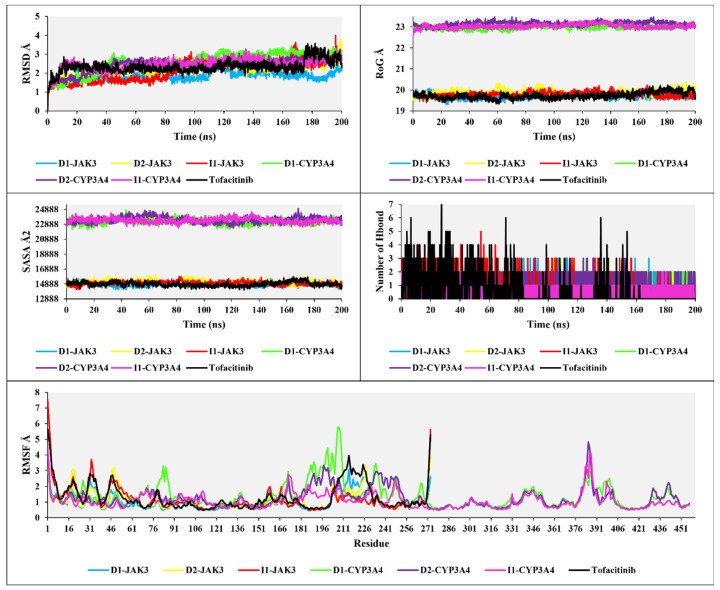
The plots of RMSD, RoG, SASA, H-bond, and RMSF analyses of new compounds with tofacitinib drug.

The analyses of RMSD, RMSF, RoG, and SASA in Figure 11 provide the following results: The RMSD analysis for the three compounds, **D1**, **D2**, and **I1**, with JAK3 and CYP3A4, shows RMSD values of 1.5 Å, 2 Å, 2.2 Å, 2.6 Å, 2.5 Å, 2.4 Å, and 2 Å, respectively. The RMSD analysis, compared to tofacitinib, demonstrates favorable stability, with some exceptions in the RMSD changes. For **D1**-JAK3, there is a fluctuation ranging from 1.5 Å to 2 Å between 115 ns and 125 ns. **D2**-JAK3 exhibits a variation between 2 Å and 3.5 Å between 190 ns and 200 ns. **I3**-JAK3 experiences an increase in RMSD between 80 ns and 100 ns, with a variation ranging from 1.5 Å to 2.5 Å, indicating stability. **D1**-CYP3A4 shows an increase between 30 ns and 40 ns, with an RMSD variation reaching up to 2.5 Å, followed by stability until 200 ns. **D2**-CYP3A4 undergoes an increase between 20 ns and 40 ns, with an RMSD variation up to 2.5 Å, followed by stability. **I1**-CYP3A4, after 5 ns, exhibits favorable stability up to 200 ns. The RMSD analyses of the compounds **D1**, **D2**, and **I1** with JAK3 and CYP3A4 reveal overall stability, indicating promising stabilities.

The analyses of RoG and SASA for the compounds with JAK3 and CYP3A4 in interaction show a stable RoG ranging from 19 to 20 Å for the compounds with JAK3 and a similarly stable RoG ranging from 23 to 23.3 Å during the 200 ns simulation. Likewise, for SASA, with JAK3 present, a stable SASA ranging from 14,985 to 15,060 Å2 is observed, while with CYP3A4, it is situated between 22,990 and 23,225 Å2. These results indicate good structural complementarity and a robust binding between the protein and the ligand. The stability of the solvent-accessible surface also suggests that the ligand is well-positioned and protected from the aqueous environment, which is generally beneficial for efficient and specific interaction with the target protein.

The H-bonds for the three compounds interacting with JAK3 and CYP3A4 reveal H-bonds ranging from a maximum of seven to a minimum of one. Compared to tofacitinib, the H-bond results for **D1**, **D2**, and **I1** in interaction with JAK3 and CYP3A4 show consistent H-bond interactions without any disruptions, with a minimum of one H-bond observed. In contrast, tofacitinib experiences H-bond disruptions between 80 ns and 200 ns. The H-bond analysis indicates stable and favorable interactions between the compounds and JAK3/CYP3A4, consistent with H-bonds throughout the simulation. This suggests that the studied compounds exhibit strong binding affinity and potential for effective interaction with the target proteins. The comparison to tofacitinib highlights the stability of the observed H-bond interactions, further supporting the potential of the studied compounds as promising candidates for further drug design and development exploration.

The analysis of RMSF for the compounds **D1**, **D2**, and **I1** in interaction with JAK3, considering 275 residues, reveals rigid flexibility and overall stability of the residues. Compared to tofacitinib, there are exceptions for certain residues where the RMSF values exceed three, indicating relatively higher fluctuation and lower stability. However, the superposition of the RMSF values suggests a generally stable RMSF pattern for the studied complexes. Furthermore, when analyzing the RMSF with 450 residues for **D1**, **D2**, and **I1** in interaction with CYP3A4, a similar stable RMSF pattern is observed compared to tofacitinib. The RMSF analysis indicates that the compounds **D1**, **D2**, and **I1**, in interaction with JAK3, exhibit rigid flexibility and overall stability of the residues. Although there are exceptions with higher RMSF values for specific residues, the superposition analysis suggests a stable RMSF pattern for the studied complexes. Additionally, when considering the interaction with CYP3A4, the compounds **D1**, **D2**, and **I1** also show a stable RMSF comparable to tofacitinib.

The comprehensive analyses of RMSD, RoG, SASA, H-bonds, and RMSF provide valuable insights into the stability and structural characteristics of **D1**, **D2**, and **I1** in interaction with JAK3 and CYP3A4. The RMSD analysis reveals overall stability with some exceptions, indicating promising stability for the studied complexes. The RoG and SASA analyses demonstrate good structural complementarity and robust binding between the proteins and ligands. The H-bond analysis indicates stable and consistent interactions, suggesting strong binding affinity. Moreover, the RMSF analysis highlights the residues’ generally stable and rigid flexibility with some exceptions. 

#### 2.9.2. PCA and FEL Analyses

**Figure 12:**
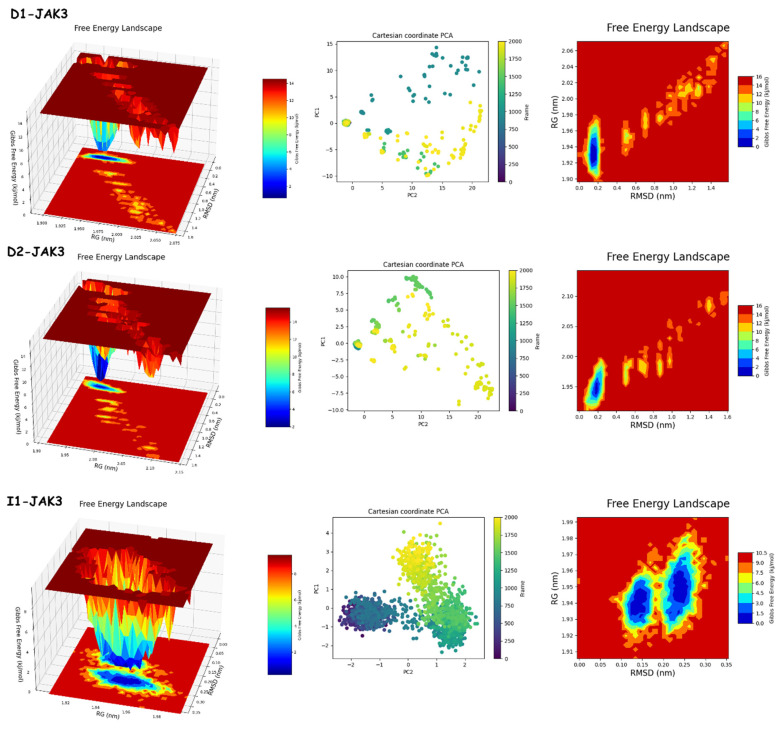
PCA and FEL analyses of new compounds.

**Figure 13:**
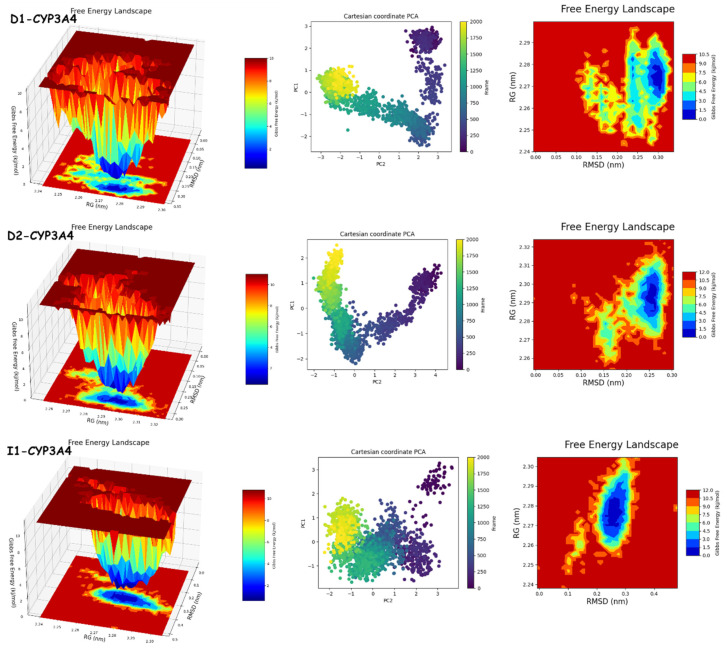
Comparative analysis of FEL and PCA graphs in 2D and 3D for compounds **D1**, **D2**, and **I1**.

The results of PCA and FEL in Figure 12 and Figure 13 provide the following. First, the PCA results are as follows: **D1**-JAK3 experienced a PCA for PC1 and PC2 ranging from −10–15 to 0–20, respectively. **D2**-JAK3 experienced a PCA for PC1 and PC2 ranging from −10–15 to 0–20, respectively. **I1**-JAK3 experienced a PCA for PC1 and PC2 ranging from −2–10 to −2–2, respectively. **D1**-CYP3A4 experienced a PCA for PC1 and PC2 ranging from −2–3 to −3–3, respectively. **D2**-CYP3A4 experienced a PCA for PC1 and PC2 ranging from −2–2 to −2–4, respectively. **I1**-CYP3A4 experienced a PCA for PC1 and PC2 ranging from −1–3 to −2–3, respectively. Secondly, FEL: **D1**-JAK3 had a stable conformation energy minimum between an RMSD of 0.19 nm and an RoG of 1.94 nm. **D2**-JAK3 had a stable conformation energy minimum between an RMSD of 0.19 nm and an RoG of 1.95 nm. **I1**-JAK3 had two stable confirmation energy minima located between an RMSD of 0.15 nm and an RoG of 1.94 nm and between an RMSD of 0.24 nm and an RoG of 1.95 nm. **D1**-CYP3A4 had two stable confirmation energy minima located between an RMSD of 0.3 nm and an RoG of 2.27 nm. **D2**-CYP3A4 had two stable confirmation energy minima located between an RMSD of 0.2209 nm and an RoG of 2.29. **I1**-CYP3A4 had two stable confirmation energy minima located between an RMSD of 0.15 nm and an RoG of 2.27 nm.

The results of the PCA analysis indicate the principal components (PC1 and PC2) for each protein (**D1**-JAK3, **D2**-JAK3, **I1**-JAK3, **D1**-CYP3A4, **D2**-CYP3A4, and **I1**-CYP3A4) and their corresponding ranges. On the other hand, the FEL analysis provides information about the stable conformation energy minima for each protein, as indicated by their respective RMSD and RoG values. These findings contribute to a better understanding of the structural characteristics and dynamics of the proteins under investigation.

**Figure 14:**
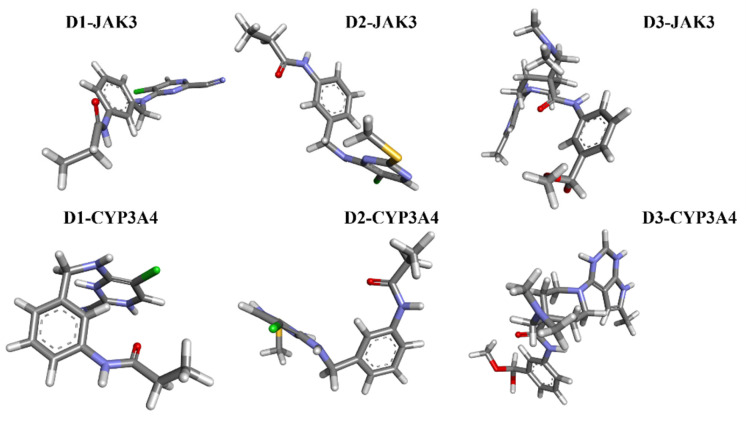
The stable conformational structure for each new compound that favors the energy minima of FEL and PCA.

The analyses of FEL and PCA allow for the extraction of stable conformations corresponding to each divergent minimum during the 200 ns simulation on the target. The results in Figure 14 suggest that the new compounds can adopt multiple stable conformations in the active site to achieve inhibition of the studied proteins, JAK3 and CYP3A4.

### 2.10. MM/GBSA Analysis

The results of MM/GBSA analysis can be used to prioritize compounds for further optimization or guide the design of new ligands with improved binding affinity. The MM/GBSA analysis is a valuable tool for understanding and predicting the binding energetics of molecular complexes, aiding in structure-based drug design and optimization.

The results of the MM/GBSA analysis show the energy contributions of different components and complexes (Table 11). Δ_TOTAL_ represents negative values that indicate favorable binding, while positive values suggest unfavorable binding. In this case, **D1**-JAK3, **D2**-JAK3, and **I1**-JAK3 show negative Δ_TOTAL_ values, indicating favorable binding to JAK3. On the other hand, **D1**-CYP3A4, **D2**-CYP3A4, and **I1**-CYP3A4 exhibit positive Δ_TOTAL_ values, suggesting less favorable binding to CYP3A4. ΔG_SOLV_ represents the change in solvation energy upon complex formation. Positive values indicate an increase in solvation energy, while negative values suggest a decrease. Notably, **I1**-JAK3 shows a significantly positive ΔG_SOLV_ value, indicating a substantial increase in solvation energy upon complex formation.

ΔG_GAS_ represents the change in the gas–phase interaction energy upon complex formation. Negative values indicate favorable interactions, while positive values suggest unfavorable interactions. In this case, **D1**-JAK3, **D2**-JAK3, and **I1**-JAK3 exhibit negative ΔG_GAS_ values, indicating favorable gas–phase interactions with JAK3. ΔE_SURF_ represents the change in the surface energy upon complex formation. Negative values suggest a decrease in surface energy, indicating favorable binding. All complexes show negative ΔE_SURF_ values, indicating favorable changes in surface energy. ΔE_GB_ represents the change in the generalized Born energy upon complex formation. Positive values indicate an increase in the generalized Born energy, while negative values suggest a decrease. Notably, **I1**-JAK3 shows a significantly positive ΔE_GB_ value, indicating a substantial increase in the generalized Born energy upon complex formation. ΔE_EL_ represents the change in the electrostatic energy upon complex formation. Negative values indicate favorable electrostatic interactions, while positive values suggest unfavorable interactions. In this case, **D1**-JAK3 and **D2**-JAK3 exhibit negative ΔE_EL_ values, indicating favorable electrostatic interactions with JAK3. Δ_VDWAALS_ represents the change in the van der Waals energy upon complex formation. Negative values indicate favorable van der Waals interactions, while positive values suggest unfavorable interactions. Notably, **D2**-CYP3A4 and **I1**-CYP3A4 show negative Δ_VDWAALS_ values, indicating favorable van der Waals interactions with CYP3A4.

## 3. Methods and Materials

### 3.1. Dataset

The dataset used in this study was collected based on previous work and included 75 inhibitors of JAK3 (Table S1, Supplementary Materials), along with their experimentally determined inhibitory biological activity values [38,39]. These biological activity values were converted into pIC_50_ using the formula −log (IC_50_ × 10^−9^). The dataset was randomly divided without any specific rule for this type of study. Fifty-nine molecules were included in the training set, with consideration given to the most active molecule in the training set. Sixteen molecules were reserved for the test set. The table below presents the predicted pIC_50_ values by both field-based and atom-based QSAR models, along with the residual errors compared to the experimental pIC_50_ values. 

### 3.2. Building Robust Models: Exploring Three-Dimensional QSAR in Development

#### 3.2.1. Preparation of Ligands

The accurate alignment of compounds is crucial for ensuring the quality and predicted activity of both the 3D-QSAR and pharmacophore models [40]. Initially, the molecular structures were in the 2D-SDF format and subsequently converted into 3D structures (Figure 5). 

The LigPrep module of Schrödinger version 2021-3 was used to prepare the ligands, ensuring the generation of high-quality structures with appropriate ionization states, tautomeric forms, ring conformations, and stereochemistry. All the molecules underwent energy minimization using the OPLS_2005 force field to optimize their structures. The alignment of the molecules was achieved using the alignment tool in Schrödinger version 2021-3 into Maestro using the Flexible Ligand Alignment Panel, which provided the capability to execute a versatile alignment for the selected entries in the Project Table. The initial selected entry functioned as a template and remained unchanged. Subsequent ligands underwent a ligand torsional search utilizing ConfGen [41]. The conformers generated by ConfGen were then sequentially aligned with the reference ligand, and the conformer exhibiting the optimal overlap with the reference ligand was selected. This chosen conformer replaced the existing entry, so it was essential to duplicate the original structures if you wished to retain them. It is recommended to employ well-minimized structures as input for flexible ligand alignment. The structures must contain hydrogens, and implicit hydrogens are not allowed [42], taking into consideration the template molecule with the highest pIC_50_ value.

#### 3.2.2. Field-Based and Atom-Based 3D-QSAR

Researchers have favored quantitative structure–activity relationships (QSARs) for optimizing lead compounds over the years. Nevertheless, conventional QSAR models usually involve only rough approximations of 3D structures. There are two methods available in Maestro for QSAR modeling: atom-based QSAR utilizes atom types and their occupancy within a grid of cubes as independent variables for fitting and predicting properties, and field-based QSAR implements the ComFA/ComSIA approach, using potential values on a grid for fitting and predicting properties. In Maestro, make your selection accordingly.

The PHASE module from Maestro, an interface of Schrödinger’s version 2021-3 utility, was utilized to develop 3D-QSAR models. To better understand the correlation between structural features and biological activity, we aimed to develop both atom-based and field-based 3D-QSAR models. The models were developed by randomly selecting a training set and a test set, following the 80:20 split recommended in the literature, and widely adopted [43,44]. However, we took steps to ensure that the developed models were not the result of random chance, and they were further evaluated for internal and external validation to determine their statistical significance. To ensure the reliability of the developed models, both active and inactive molecules were included in both the training and test sets. The same strategy was used for MLR-based QSAR models, and in all cases, we thoroughly assessed the robustness of our models. The dataset was randomly divided into an 80% training set and a 20% test set, with a PLS factor of 4 applied to both 3D-QSAR models. The random selection made by software was visually verified to ensure diversity among the molecules in the training and test sets. We maintained a 1 Å grid spacing for the selected hypothesis. We developed four atom-based 3D-QSAR models and four field-based models (Table 2, Table 3, Table 4, Table 5 and Table 12). For both the field-based and atom-based models, the training set consisted of 75 molecules, while the test set consisted of 13 molecules (Table S1). The Gaussian field-based 3D-QSAR models incorporated descriptors such as Gaussian steric, electrostatic, hydrophobic, hydrogen bond donor, and hydrogen bond acceptor. In the field-based models, Gaussian intensities were considered as independent variables. Finally, the best-selected 3D-QSAR models were developed to visualize the 3D contour maps associated with structural features (Figure 1). The visualization of QSAR models is important for optimizing the scaffolds.

#### 3.2.3. Evaluating the Predictive Power of 3D-QSAR Models: A Comparative Analysis

The essential metrics used for evaluating three-dimensional quantitative structure–activity relationship (3D-QSAR) models will be explored. These metrics provide a crucial insight into the quality and reliability of models, guiding the compound optimization process. R^2^ (coefficient of determination): The proportion of the variance in the dependent variable explained by the independent variables is measured. An optimal fit is indicated by an R^2^ close to R^2^_CV_ (cross-validated coefficient of determination): As with R^2^, this is calculated using cross-validation methods, assessing the model’s ability to generalize to independent data. RMSE (root mean square error): The average of errors between predicted and observed values is indicated, providing an overall measure of model accuracy. Q^2^ (cross-validated coefficient of determination): Similar to R^2^_CV_, it evaluates how well the model predicts new, unseen data using cross-validation methods. These metrics, when employed, offer a comprehensive understanding of the predictive power and reliability of 3D-QSAR models, contributing to informed decision making in compound design and optimization.

The evaluation of the 3D-QSAR model involved the assessment of key statistical parameters, including the squared cross-validation coefficient (Q^2^), squared non-cross-validation coefficient (R^2^), predictive R^2^, and standard error of estimate (SEE). To determine the internal quality of the developed model, the Q^2^ value was considered, with a criterion of >0.5 deemed statistically significant. The R^2^ value was utilized as a relative measure of the regression fit, with a value close to 1.0 indicating a solid fit. Additionally, the standard error of estimate provided insights into the variation in residuals or the regression line [45,46].

### 3.3. Pharmacophore Hypothesis Generation

The pharmacophore hypothesis is a widely used approach in pharmaceutical chemistry that aims to identify and model the essential interactions between a drug molecule and its biological target. This method is based on the understanding that specific structural or chemical characteristics of the molecule are crucial for its biological activity [12,47,48]. Advanced Schrödinger software version 2021 provides sophisticated tools for generating and validating pharmacophore hypotheses by utilizing information on molecular interactions, such as hydrogen bonds, electrostatic interactions, and hydrophobic interactions [42].

To prepare the structure data file for our test compounds, we utilized the LigPrep panel integrated within Schrödinger software version 2021-3. The ligand chemistry was appropriately normalized and extrapolated for pharmacophore modeling using PHASE, an automated process that aligns the ligands based on their optimal arrangement and shared properties (Figure 5B). The geometrical optimization through condensed Newton conjugate gradient (TNCG) minimization employed the OPLS_2005 force field. Ligands were prepared using LigPrep (Schrödinger version 2021-3) with Epik, adjusting the pH to 7 ± 2.0 units to account for protonation and tautomeric states and utilizing the OPLS_2005 force field. Subsequently, the prepared ligands were imported into the Maestro workspace, and their experimental binding affinities (pIC_50_) were used to categorize them as active or inactive. The pIC_50_ values were calculated using the equation pIC_50_ = −log (IC_50_), where an IC_50_ affinity of ≤50 nM corresponded to a pIC_50_ value greater than 7.5. Inactive molecules were identified using a threshold of 10 µM or a pIC_50_ value below 7.5. The assumption requirement was set to match at least 50% of the active compounds, and a minimum of five features were preferred for a successful match. The assumption difference criteria remained at their default settings, except for donor and negative molecules, where ionic features were assigned a value of 1 to ensure compatibility between the acceptor and negative features.

### 3.4. Molecular Docking

Irreversible (covalent) docking and reversible (non-covalent) docking are two distinct approaches used in the field of molecular docking, a computational technique employed to predict the binding affinity and orientation of a small molecule (ligand) within a receptor or target protein [49].

#### Irreversible (Covalent Docking)

Irreversible (covalent) docking involves the formation of a covalent bond between the ligand and the target protein. In this approach, the ligand is designed to contain a reactive functional group that can form a stable covalent bond with specific amino acid residue in the target protein, typically a nucleophilic residue such as a cysteine or a serine. Once the covalent bond is formed, it is generally irreversible, meaning that the ligand cannot easily dissociate from the target protein. Irreversible docking is often used when designing drugs that require long-lasting or irreversible inhibition of their target proteins [50,51].

### 3.5. Protein Structure Preparation

The protein structures of JAK3 (PDB ID: 4Z16) were obtained from the RCSB Protein Data Bank. They were imported into the Maestro program for further processing using the Protein Preparation Wizard in Schrödinger software. The aim was to optimize the protein structure, maximize H-bond interactions of side chains, and perform energy minimization using the OPLS_2005 force field [52]. In the case of the model protein 4Z16, the ligands were covalently bound to Cys909. Two approaches were taken for treatment: one involved disconnecting the co-crystallized ligand from Cys909, minimizing the protein, and saving it as a non-covalent model; the other involved maintaining the covalent bond between the ligand and Cys909 during minimization for covalent docking.

The selection of proteins for this study is driven by several factors: superior resolution compared to other proteins, absence of mutations, and a more favorable alignment of the ligand structure with experimental data. Specifically, JAK3 (4Z16) was employed in the research due to its distinction as the first protein featuring a co-crystallized ligand containing an acrylaldehyde group. This structural attribute facilitates the formation of a covalent bond with Cys909, effectively inhibiting JAK3. The decision to use JAK3 (ID: 4Z16) is rooted in its recognition as one of the primary proteins capable of elucidating the presence of the covalent bond between JAK3 and the Cys909 residue. Moreover, the co-crystallized ligand in the study is identified as an EGFR inhibitor, underscoring its efficacy in treating autoimmune diseases. Furthermore, JAK3 (ID: 4Z16) is acknowledged as one of the target proteins with superior resolution compared to other types found on UniProt and PDB. The requisite metrics are also available on PDB, and JAK3 (ID: 4Z16) does not manifest any mutations. Similarly, for CYP3A4 (ID: 5VCC), an in-depth search was conducted among proteins that could yield desired results with high resolution and the metrics available on PDB, taking into consideration the presence of mutation(s). 

### 3.6. Schrödinger Covalent Docking

To study the effect of covalent docking, ligand JAK3 inhibitors were docked to the model protein 4Z16 using the covalent dock program within Schrödinger software suite. The setup process involved specifying Michael addition or Ketone-Cysteine as the reaction type for the new compounds designed, and the scoring function was set to Extra Precision. Default parameters were used for all other settings.

#### Reversible (Non-Covalent)

Reversible (non-covalent) docking involves the prediction of the non-covalent interactions between the ligand and the target protein. These interactions can include hydrogen bonding, van der Waals forces, hydrophobic interactions, and electrostatic interactions. Reversible docking predicts the most favorable binding pose and affinity of the ligand within the target protein without the formation of a covalent bond. Reversible docking is widely used in drug discovery and virtual screening to identify potential lead compounds that can bind to the target protein with high affinity and specificity. One of the advantages of reversible docking is the possibility of ligand dissociation, which allows for the development of drugs with desirable pharmacokinetic properties and reduced toxicity risks.

Before conducting molecular docking, the ligands intended for docking were optimized using Avogadro software 2.0. Subsequently, we obtained the structures of JAK3 and CYP3A4 from the RCSB database (PDB ID:4Z16 and 5VCC). The crystal complex of 4Z16 comprises water molecules and the co-crystallized ligand 4LH bound to the protein 4Z16. To prepare the protein, we eliminated all water molecules and 4LH and added polar hydrogens to the JAK3 protein structure using Discovery Studio software 2021. Similar steps were followed for 5VCC, except that the included ligands were not deleted because they are an important part of the metabolism of CYP3A4. After preparing the ligands and protein, molecular docking was carried out using AD4 and AutoVina to explore the active site of 4Z16 and 5VCC, which is determined by the region encompassing the co-crystallized ligands (4LH and HEM) [53]. The three-dimensional grid was established using the AUTOGRID algorithm, which calculates the binding energy between ligands and their receptor. The default grid size for JAK3 with tofacitinib and CYP3A4 with new compounds was set to x = 60, y = 60, and z = 60, with a spacing of 0.375 Å between grid points. The center of the grid corresponds to the active site of the receptors JAK3-4LH and CYP3A4 HEM, with coordinates (x = −6.68875 Å, y = −14.7757 Å, and z = 1.89597 Å) and (x = −19.3381 Å, y = −30.475 Å, and z = 17.7174 Å), respectively. The docking results obtained from AD4 and Vina were visualized using Discovery Studio software 2021.

### 3.7. Predictive Toxicity Analysis and Bioactivity Assessment

ADMET analysis, medicinal chemistry, and the assessment of lead-like and drug-like properties were conducted using readily accessible online tools. These tools, such as SwissADME (http://www.swissadme.ch/ (accessed on 22 April 2022)) [54] and pkCSM (http://structure.bioc.cam.ac.uk/pkcsm (accessed on 23 April 2022)) [36], evaluate drug candidates and compounds to determine their potential toxicity for human use.

### 3.8. Molecular Dynamics Simulation

The newly created compounds, which exhibited enhanced binding affinity with JAK3 and CYP3A4, underwent all-atom molecular dynamics simulations using GROMACS 2021 (Groningen Machine for Chemical Simulation) software [55,56]. Before initiating the MD simulations, the CHARMM-GUI web server [57] was employed to generate the initial input parameters, implementing the CHARMM36 force field [58,59,60,61]. The simulation was conducted at a pH of 7. Before entering the production phase, each complex was solved within a rectangular grid box, surrounded by TIP3P water molecules, and supplemented with the requisite counter-ions (Na^+^, Cl^−^) to maintain a salt concentration of 0.15 M, achieved through Monte Carlo ion displacement. Energy minimization was executed for each system using the steepest descent algorithm, encompassing a maximum of 50,000 steps and a maximum force of 10.0 kJ/mol. The temperature and atmospheric pressure were set to 310 K and 1.01325 bar, respectively. For NVT equilibration, two stages were carried out, each lasting 10 ns. Canonical (NVT) and isothermal-isobaric (NPT) ensembles were utilized for equilibrating each system. Subsequently, MD simulations were conducted for a duration of 200 nanoseconds. To assess the structural stability of the designed molecules, various parameters, including root mean square deviation (RMSD), the radius of gyration (RoG), solvent accessible surface area (SASA), and root mean square flexibility (RMSF), were analyzed based on the dynamics trajectory results.

### 3.9. Evaluating Binding Free Energy with Molecular Mechanics/Generalized Born Surface Area (MM/GBSA)

The Molecular Mechanics/Generalized Born Surface Area (MM/GBSA) method is an efficient force field technique employed to evaluate the binding free energy of a system, specifically in kcal/mol [62]. For more information, free binding energy ([12]) can be visited. These calculations were conducted by utilizing the last 200 frames and were determined through the application of the following equations.

ΔG_bind_ = G_complex_ − G_protein_ − G_ligand_ (1)

ΔG_bind_ = ΔG_gas_ + ΔG_sol_ − TΔS (2)

ΔG_gas_ = Bond + Angle + Dihed + EEL + VdW (3)

ΔG_sol_ = ΔE_GB_ + ΔE_SURF_ (4)

ΔG_bind_ represents the total binding free energy of the system, as elucidated in Equation (1), and is determined through the utilization of Equation (2). TΔS denotes the change in conformational entropy that arises from the binding of the ligand at a specific temperature. ΔG_gas_ encompasses the combined contributions stemming from bond, angle, dihedral, EEL (the electrostatic element of internal energy), and van der Waals energies, as expounded in Equation (3). The internal energy relates to the oscillations and rotations of individual bond torsional angles. Solvation-free energy (ΔG_sol_) is composed of both ΔE_GB_ (the polar component of solvation energy) and ΔE_SURF_ (the nonpolar component of solvation energy), as detailed in Equation (4).

## 4. Conclusions

In conclusion, this 3D-QSAR study utilizing field-based and atom-based approaches and the DHRRR pharmacophore model has provided valuable insights into the design and identification of JAK3 and CYP3A4 inhibitor compounds. The favorable validation results have instilled confidence in using these models for in silico prediction of compound activity. The study has led to the discovery of novel compounds by applying 3D-QSAR on a collected series of molecules with known activity. Screening of a database of molecules, based on their inhibitory activity against JAK3, has resulted in identifying three hits exhibiting strong affinity and favorable non-toxicity. These hits were further evaluated using molecular docking and ADMET analysis to assess their pharmaceutical properties. To validate the potential of these compounds, confirmation was sought through MD simulation and MM/GBSA analysis. These simulations’ results support the identified compounds’ promising nature, indicating their potential as candidates for drug design and development. These findings highlight the importance of utilizing 3D-QSAR techniques in designing novel compounds, which can lead to the discovery of potent inhibitors. Further exploration and experimental validation are warranted to fully exploit the potential of these compounds and progress them toward becoming viable drug candidates.

## Figures and Tables

**Table 1:** Advantages and disadvantages of the α,β-Unsaturated ketones and Michael 1,4 reaction.

Formation of a Covalent Bond with α,β-Unsaturated Ketone:	
**Advantages**	**Disadvantages**
1. Wide applicability	1. Limited reactivity of some α,β-unsaturated ketones
2. Generally efficient reaction	2. Incompatibility with certain functional groups
3. Structural diversity	3. Formation of undesired by-products
**Michael 1,4 Reaction:**	
**Advantages**	**Disadvantages**
1. Formation of C-C bonds	1. Limited reactivity of some nucleophiles
2. Simple reaction steps	2. Competitive side reactions
3. Wide range of acceptor enones	3. Sensitivity to steric effects

**Table 2:** The statistical data for the field-based QSAR model.

Factors	SD	R^2^	R^2^_CV_	R^2^ Scramble	Stability	F	P	RMSE	Q^2^	Pearson-r
1	0.7667	0.4738	0.3882	0.1235	0.99	44.1	2.38 × 10^−8^	0.59	0.5667	0.8013
2	0.6364	0.6449	0.4337	0.2443	0.936	43.6	1.62 × 10^−11^	0.52	0.6729	0.8383
3	0.5389	0.7506	0.4161	0.3818	0.818	47.2	3.24 × 10^−14^	0.52	0.6686	0.8181
4	0.5023	0.7880	0.5334	0.4656	0.805	42.7	6.13 × 10^−15^	0.44	0.7630	0.8775

**Table 3:** The statistical data for the atom-based QSAR model.

Factors	SD	R^2^	R^2^_CV_	R^2^ Scramble	Stability	F	P	RMSE	Q^2^	Pearson-r
1	0.6463	0.5585	0.47	0.1595	0.987	62	3.00 × 10^−10^	1.04	0.2246	0.4915
2	0.535	0.7037	0.5033	0.2836	0.909	57	2.10 × 10^−13^	0.91	0.4079	0.6392
3	0.4215	0.8199	0.4942	0.4679	0.810	71.3	1.61 × 10^−17^	0.77	0.5738	0.7576
4	0.3533	0.8761	0.5683	0.5665	0.655	81.3	2.91 × 10^−20^	0.69	0.6633	0.818

**Table 4:** Statistical analysis of atom-based model in 3D-QSAR.

Factors	Steric	Electrostatic	Hydrophobic	H-Bond Acceptor	H-Bond Donor
1	0.41	0.115	0.149	0.261	0.066
2	0.287	0.095	0.223	0.29	0.105
3	0.319	0.122	0.219	0.19	0.149
4	0.333	0.125	0.209	0.184	0.149

**Table 5:** Newly designed compounds with their predicted pIC_50_ for QSAR models.

Compound	2D	Affinity (Kcal/mol)	pIC_50_ (Pred) Field-Based	pIC_50_ (Pred) Atom-Based
**D1**	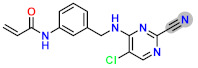	−8.67	8.42353	7.75
**D2**	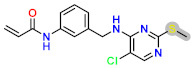	−8.18	7.47945	7.00
**D3**	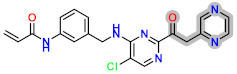	−7.48	8.34337	8.30
**D4**	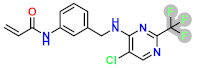	−8.56	8.28205	7.94
**D5**	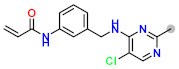	−7.87	8.01863	7.48
**D6**	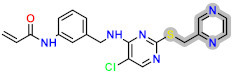	−7.77	8.0394	7.54
**D7**	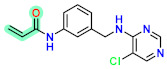	−7.38	8.42353	7.75

**Table 6:** Various pharmacophore hypotheses were generated by utilizing the compounds and their corresponding activities.

Model	Survival Score	Site Score	Vector Score	Volume Score	Score	PhaseHypoScore
DHRRR_2	6.102	0.776	0.908	0.754	0.973	1.339
DHRRR_1	6.168	0.794	0.927	0.777	0.972	1.342
DHRR_2	5.773	0.861	0.954	0.761	0.804	1.150
DHRR_1	5.813	0.897	0.973	0.765	0.961	1.310
DDRRR_2	5.985	0.764	0.933	0.793	0.948	1.307
DDRRR_1	6.033	0.792	0.929	0.812	0.956	1.318
DDHRR_2	6.225	0.825	0.963	0.737	0.945	1.318
DDHRR_1	6.265	0.837	0.951	0.765	0.932	1.308
DDHR_1	5.772	0.895	0.948	0.760	0.765	1.111
AHRR_1	5.742	0.895	0.977	0.729	0.812	1.156
ADRR_1	5.674	0.941	0.975	0.782	0.950	1.290
ADHRR_3	6.003	0.751	0.913	0.730	0.886	1.246
ADHRR_2	6.152	0.844	0.979	0.729	0.906	1.276
ADHRR_1	6.255	0.904	0.983	0.743	0.948	1.324
ADHR_3	5.665	0.885	0.951	0.722	0.920	1.260
ADHR_2	5.717	0.901	0.957	0.726	0.815	1.158
ADDHR_1	5.980	0.845	0.956	0.777	0.928	1.286

**Table 7:** Evaluation metrics for hypothesis DHRR_1.

Hypothesis	PhaseHypoScore	EF1%	BEDROC160.9		Matches	
DHRR_1	1.3	1.31	1		4 of 4	
BEDROC:1						
alpha*Ra			alpha			
160.9,			122.5905			
BEDROC: 0.940						
alpha*Ra			alpha			
15.2381			20.0			
BEDROC: 0.874						
alpha*Ra			alpha			
6.0952			8.0			
ROC			0.66			
Count and percentage of actives in top N% of decoy results.						
% Decoys		1%	2%	5%	10%	20%
# Actives		0	0	0	5	19
% Actives						
Count and percentage of actives in top N% of results.						
% Results		1%	2%	5%	10%	15%
# Actives		0	1	3	5	11
% Actives		0	2.1	6.2	10.4	11
Enrichment factors concerning N% sample size.						
% Sample		0.01	0.02	0.05	0.1	0.2
EF		1.3	1.3	1.3	1.1	1.2
EF*		inf	inf	1.6	2	2
EF’		inf	inf	1.6	2.5	2.5
DEF		n/a	n/a	n/a	n/a	n/a
DEF*		n/a	n/a	n/a	n/a	n/a
DEF’		n/a	n/a	n/a	n/a	n/a
Eff		−1	−1	−1	0.0204	0.329
Enrichment factors concerning N% actives recovered.						
% Actives		40%	50%	60%	70%	80%
EF		1.1	1.1	1.1	1.1	1.1
EF*		3	1.5	1.3	1.3	1.3
EF’		2.5	2.1	1.9	1.7	1.7
FOD		0.07	0.1	0.2	0.2	0.3

EF (Enrichment Factor): it represents the absolute enrichment factor of the model. It corresponds to the ratio between the active fraction in the selected model and the known active fraction in the entire chemical library. The higher it is, the better the model. EF’ (Normalized Enrichment Factor): it normalizes the EF relative to the expected random enrichment given the sample size and the rate of actives in the chemical library. It is the most significant factor for comparing models to each other. EF* (Robust Enrichment Factor): it integrates the statistical uncertainty related to the sample size in the enrichment calculation. It is therefore more robust when samples are small. DEF* (Diverse Robust Enrichment Factor): similar to EF*, it integrates the uncertainty related to the sample size. The difference is that it considers chemical diversity (scaffold, physico-chemical properties) within the hits. It therefore allows evaluating the enrichment in terms of chemical diversity. DEF’ (Normalized Diverse Enrichment Factor): analogous to DEF*, it normalizes DEF relative to the expected random enrichment given the N% sample size, the rate of actives in the chemical library and the chemical diversity.

**Table 8:** Newly identified compounds with their predicted pIC_50_ for QSAR models.

Compounds	ID	Affinity (kcal/mol)	Smiles	pIC_50_ (Pred) Field-Based	pIC_50_ (Pred) Atom-Based
**I1**	BDBM50117501	−8.72	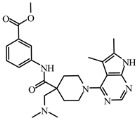	8.51	7.98
**I2**	SCHEMBL20184389	−5.63	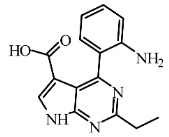	7.16	7.49
**I3**	SCHEMBL645138	−8.62	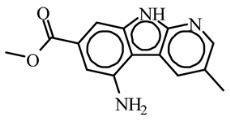	7.19	6.90
**I4**	BDBM50117502	−8.52	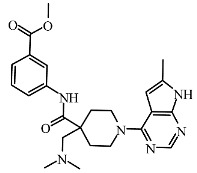	8.46	7.88
**I6**	SCHEMBL5253185	−7.80	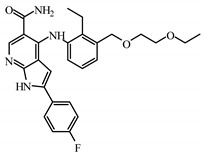	8.60	7.99
**I7**	ZGEWVZMORUOOMV	−6.85	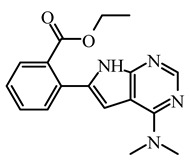	8.03	7.60
**I8**	60118026	−7.93	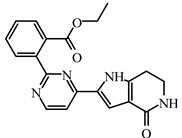	7.38	7.23

**Table 9:** ADMET evaluation of newly designed compounds.

Compound	Adsorption		Distribution	Metabolism							Excretion	Toxicity
	Water Solubility (Logs)	Intestinal Absorption (Human)	Permeability	Substrate		Inhibitor						
			BBB	2D6	3A4	1A2	2C19	2C9	2D6	3A4	Total Clearance	AMES Toxicity
**D1**	−3.376	76.324	−0.726	No	Yes	No	No	No	No	Yes	0.078	No
**D2**	−3.931	88.657	−0.796	No	Yes	No	Yes	Yes	No	Yes	0.02	No
**D3**	−4.456	89.197	−0.024	No	Yes	Yes	Yes	Yes	No	No	−0.057	No
**D4**	−3.825	92.308	−0.007	No	Yes	Yes	Yes	No	No	No	−0.059	No
**D5**	−3.748	85.635	−0.764	No	Yes	No	Yes	Yes	No	Yes	0.19	No
**D6**	−3.599	92.597	0.146	No	Yes	Yes	Yes	No	No	No	−0.004	No

**Table 10:** ADMET assessment of newly identified molecules.

Compound	Adsorption		Distribution	Metabolism							Excretion	Toxicity
	Water Solubility (Logs)	Intestinal Absorption (Human)	Permeability	Substrate		Inhibitor						
			BBB	2D6	3A4	1A2	2C19	2C9	2D6	3A4	Total Clearance	AMES Toxicity
**I1**	−3.055	81.075	−1.028	No	Yes	No	No	No	No	Yes	0.589	No
**I2**	−2.889	60.562	−1.433	No	No	No	No	No	No	No	0.709	No
**I3**	−2.919	95.844	−1.132	No	No	Yes	No	No	No	No	0.62	Yes
**I4**	−3.055	81.075	−1.028	No	Yes	No	No	No	No	Yes	0.589	No
**I5**	−3.084	81.619	−1.022	No	Yes	No	No	No	No	Yes	0.598	No
**I6**	−4.614	89.092	−1.017	No	Yes	No	Yes	Yes	No	Yes	0.477	Yes
**I7**	−2.899	97.033	−0.104	No	No	Yes	Yes	No	No	No	0.931	No
**I8**	−2.995	92.516	−0.859	No	Yes	Yes	No	No	No	Yes	0.834	No

**Table 11:** Energy contributions and binding characteristics of complexes.

Delta Energy Component (Kcal/mol)	D1-JAK3	D2-JAK3	I1-JAK3	D1-CYP3A4	D2-CYP3A4	I1-CYP3A4	Tofacitinib-JAK3
Δ_TOTAL_	−21.60	−25.96	−36.51	6.53	−15.83	−29.68	−3.20
ΔG_SOLV_	33.97	26.49	110.50	−63.87	56.84	−14.72	52.55
ΔG_GAS_	−55.57	−52.45	−147.00	70.40	−72.67	−14.96	−55.75
ΔE_SURF_	−4.94	−5.18	−5.88	−4.63	−4.23	−5.77	−3.34
ΔE_GB_	38.91	31.67	116.38	−59.24	61.07	−8.95	55.89
ΔE_EL_	−20.91	−14.37	−109.57	103.24	−46.97	22.66	−32.93
Δ_VDWAALS_	−34.66	−38.08	−37.44	−32.84	56.84	−37.62	−22.82

**Table 12:** Statistical analysis of field-based model in 3D-QSAR.

Factors	H-Bond Donor	Hydrophobic/Nonpolar	Electron-Withdrawing
1	0.031	0.63	0.338
2	0.035	0.598	0.366
3	0.043	0.575	0.383
4	0.053	0.555	0.392

## Data Availability

Data are contained within the article and Supplementary Materials.

## Supplementary Materials

The following supporting information can be downloaded at: https://www.mdpi.com/article/10.3390/molecules29010023/s1, Table S1. Activity Experimental, Prediction, and Error Analysis for Series of Cys909 Linkage Agents.
