# Supplementary material for: Computational 3D Modeling-Based Identification of Inhibitors Targeting Cysteine Covalent Bond Catalysts for JAK3 and CYP3A4 Enzymes in the Treatment of Rheumatoid Arthritis
Source: Molecules. 2023 Dec 19;29(1):23. doi: 10.3390/molecules29010023 (PMC10779482; doi:10.3390/molecules29010023)
Supplement: Supplementary file 1 [file molecules-29-00023-s001.zip › molecules-2710972-supplementary.pdf]

Table S1 Activity Experimental, Prediction, and Error Analysis for Series of Cys909 Linkage Agents.

Compound	2D structure	Factor	QSAR Set	pIC_{50}	pIC_{50}(A)	Residual (A)	pIC_{50}(B)	Residual (B)
1	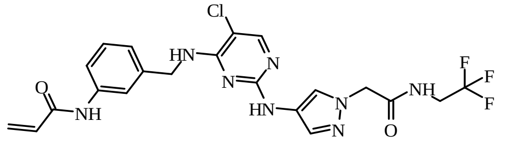	4	test	9.30	9.23	-0.07	9.32	0.02
2	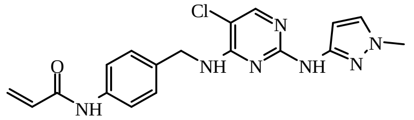	4	training	9.24	8.96	-0.28	9.21	-0.03
3	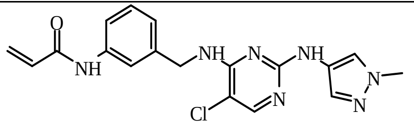	4	test	9.22	8.90	-0.32	8.93	-0.29
4	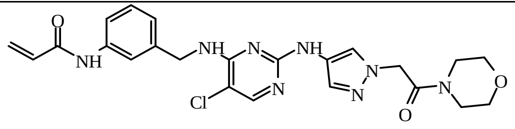	4	training	9.22	9.31	0.09	9.30	0.08
5	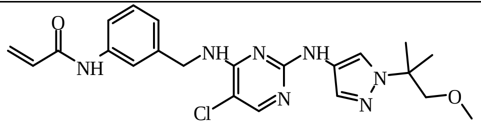	4	training	9.22	9.04	-0.18	9.38	0.16
6	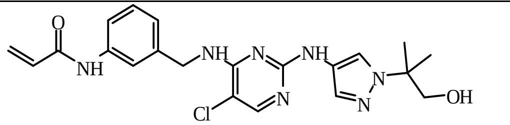	4	test	9.22	8.92	-0.30	9.28	0.06
7	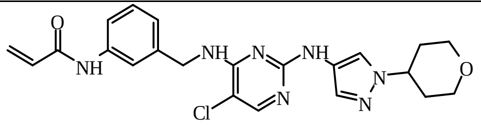	4	training	9.15	9.06	-0.09	9.26	0.11
8	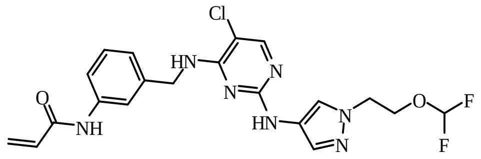	4	training	9.15	8.90	-0.25	9.10	-0.05
9	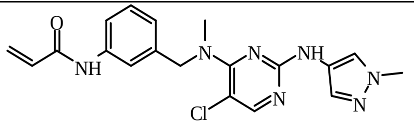	4	training	9.15	9.12	-0.03	8.88	-0.27

10	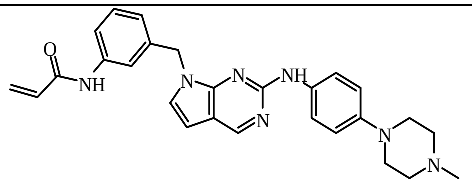	4	training	9.15	8.11	-1.04	8.74	-0.41
11	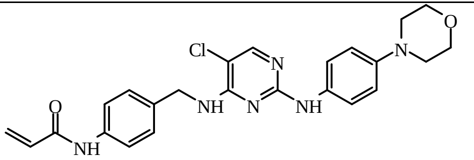	4	test	9.06	8.27	-0.79	8.87	-0.19
12	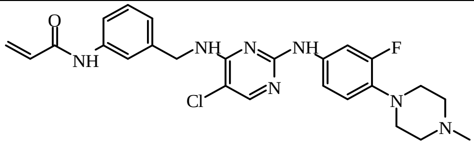	4	training	9.05	8.40	-0.65	8.32	-0.73
13	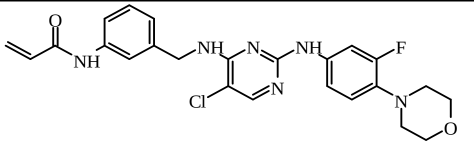	4	training	9.05	8.55	-0.50	8.31	-0.74
14	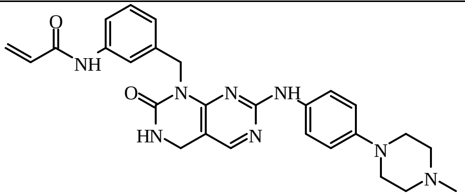	4	training	9.05	9.15	0.10	9.30	0.25
15	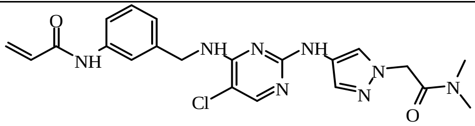	4	training	8.96	9.02	0.06	9.04	0.08
16	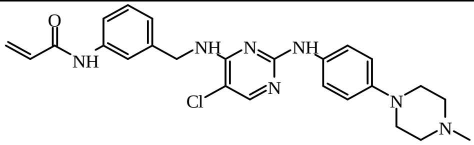	4	training	8.92	8.53	-0.39	8.27	-0.65
17	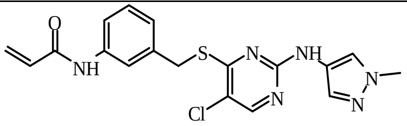	4	test	8.92	9.10	0.18	8.88	-0.04
18	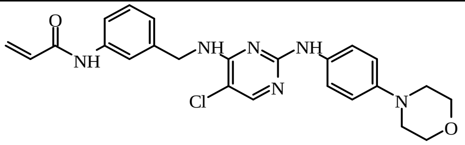	4	training	8.89	8.64	-0.25	8.37	-0.52

19	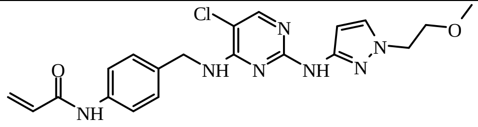	4	training	8.89	9.05	0.16	9.37	0.48
20	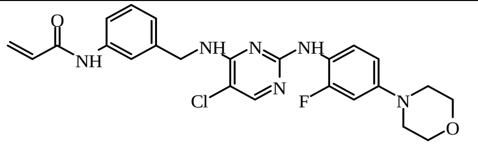	4	training	8.85	8.50	-0.35	8.45	-0.40
21	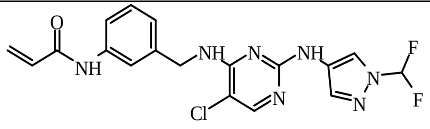	4	test	8.85	8.75	-0.10	9.01	0.16
22	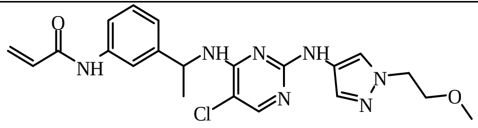	4	training	8.80	9.02	0.22	9.13	0.33
23	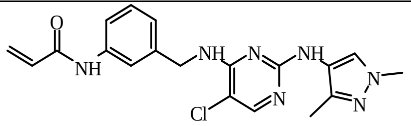	4	training	8.77	8.84	0.07	8.88	0.11
24	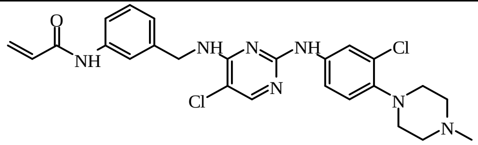	4	training	8.70	8.40	-0.30	8.37	-0.33
25	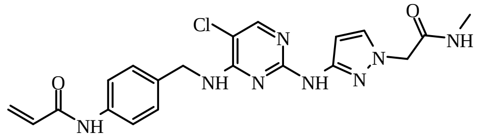	4	training	8.70	8.96	0.26	9.48	0.78
26	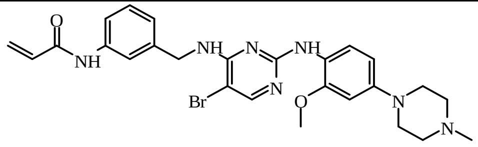	4	test	8.64	7.57	-1.07	7.65	-0.99
27	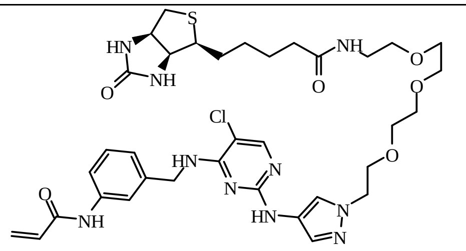	4	training	8.64	8.20	-0.44	8.77	0.13

28	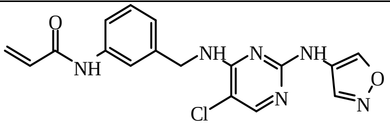	4	training	8.54	8.65	0.11	8.59	0.05
29	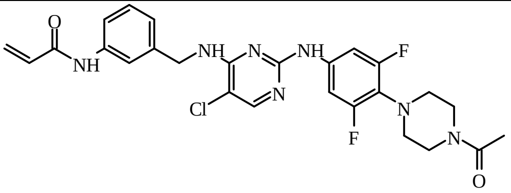	4	training	8.44	8.40	-0.04	8.30	-0.14
30	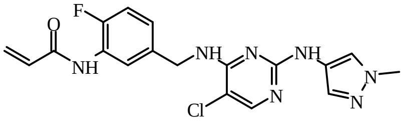	4	training	8.36	8.76	0.40	8.88	0.52
31	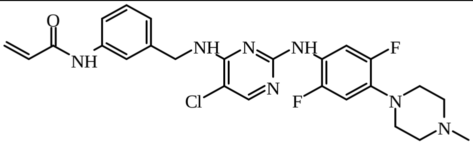	4	test	8.34	8.40	0.06	8.34	0.00
32	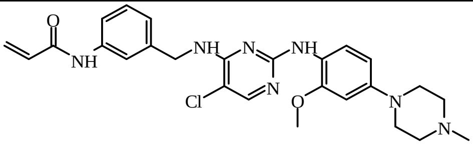	4	training	8.32	7.51	-0.81	7.53	-0.79
33	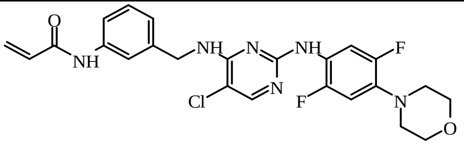	4	training	8.28	8.37	0.09	8.44	0.16
34	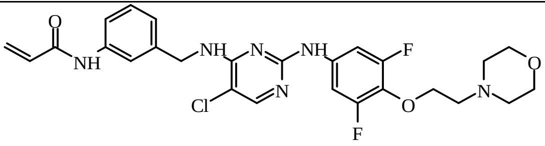	4	training	8.21	8.53	0.32	8.37	0.16
35	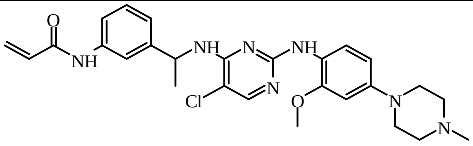	4	training	8.18	7.64	-0.54	7.74	-0.44
36	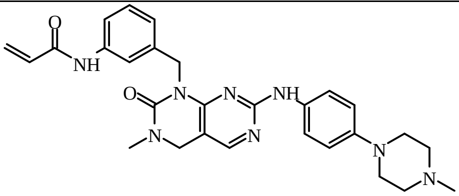	4	training	8.15	8.33	0.18	8.07	-0.08

37	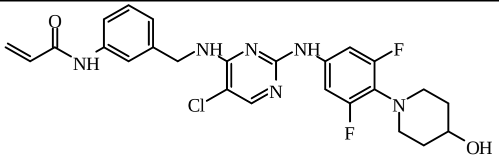	4	training	8.13	8.46	0.33	8.10	-0.03
38	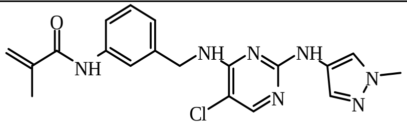	4	test	8.11	8.76	0.65	8.05	-0.06
39	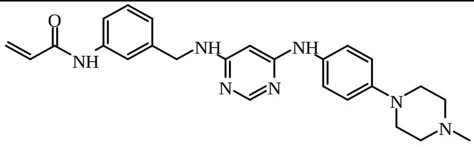	4	training	8.00	7.78	-0.22	7.52	-0.48
40	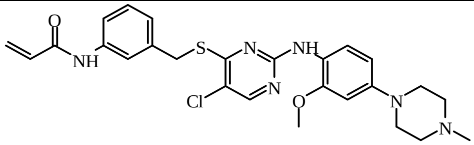	4	training	7.92	7.79	-0.13	7.53	-0.39
41	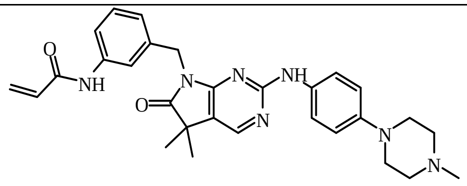	4	test	7.77	8.04	0.27	7.63	-0.14
42	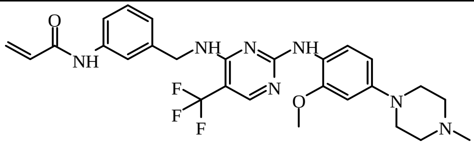	4	training	7.74	7.60	-0.14	7.53	-0.21
43	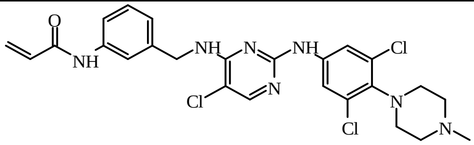	4	training	7.70	8.33	0.63	8.12	0.42
44	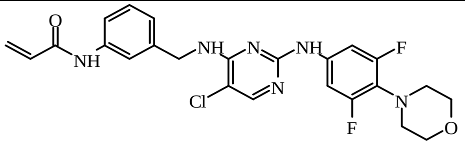	4	training	7.70	8.48	0.78	8.29	0.59
45	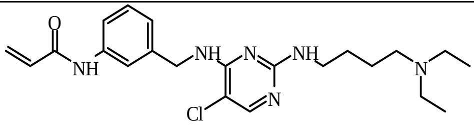	4	training	7.62	7.65	0.03	7.23	-0.39

46	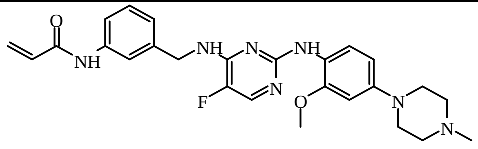	4	test	7.52	7.30	-0.22	7.17	-0.35
47	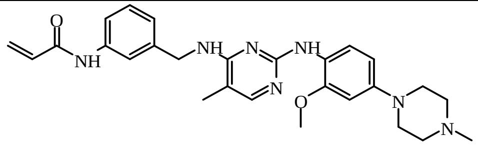	4	training	7.51	7.16	-0.35	7.53	0.02
48	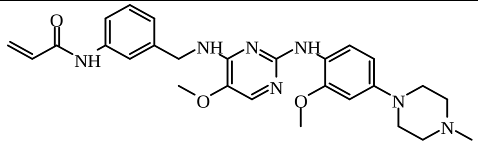	4	training	7.51	6.88	-0.63	6.92	-0.59
49	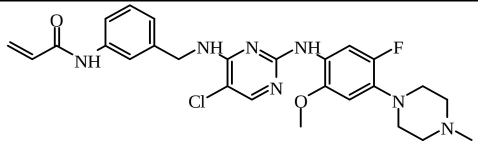	4	training	7.34	7.44	0.10	7.52	0.18
50	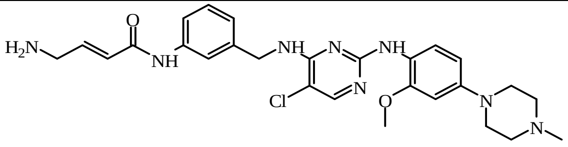	4	training	7.19	7.18	-0.01	7.05	-0.14
51	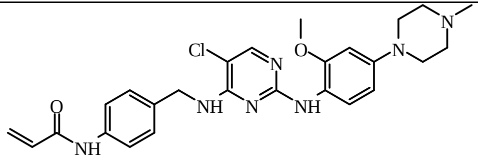	4	training	7.08	7.01	-0.07	6.87	-0.21
52	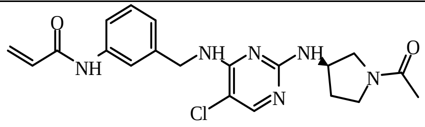	4	test	7.00	6.35	-0.65	6.72	-0.28
53	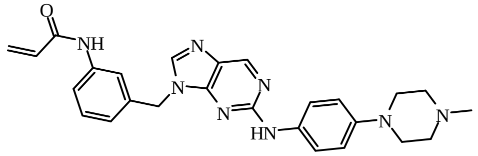	4	training	7.00	8.05	1.05	7.26	0.26
54	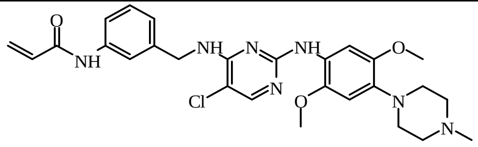	4	training	6.77	7.52	0.75	7.25	0.48

55	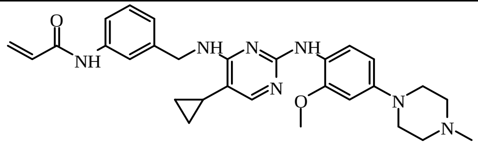	4	test	6.66	6.78	0.12	7.53	0.87		
56	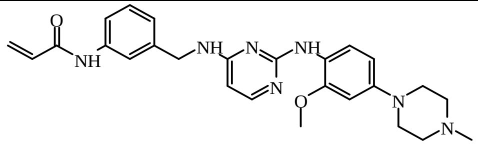	4	training	6.65	7.36	0.71	7.03	0.38		
57	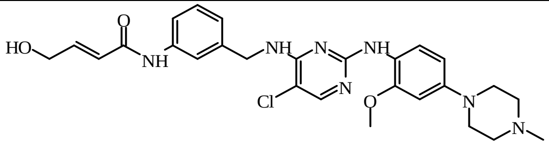	4	training	6.61	7.28	0.67	6.82	0.21		
58	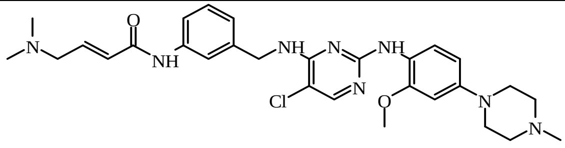	4	training	6.59	6.55	-0.04	6.78	0.19		
59	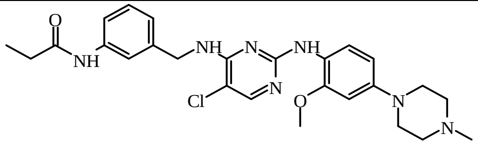	4	test	6.41	7.45	1.04	6.90	0.49		
60	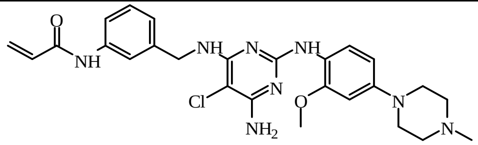	4	training	6.30	6.02	-0.28	6.55	0.25		
61	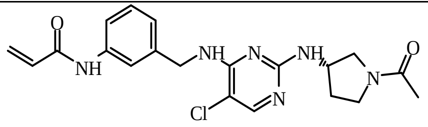	4	training	5.80	6.41	0.61	6.75	0.95		
62	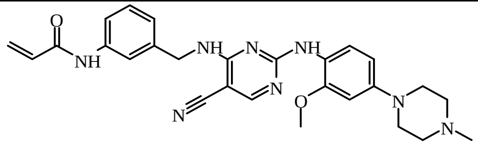	4	training	5.59	6.68	1.09	6.08	0.49		
63	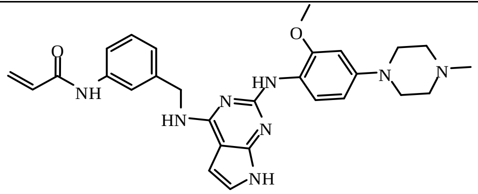	4	training	5.57	5.67	0.10	6.68	1.11		

64	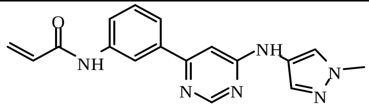	4	training	6.52	8.56	-0.49	8.56	-0.49
65	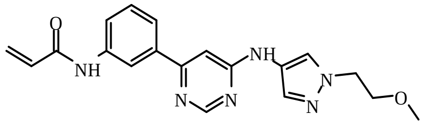	4	test	7.00	8.45	-0.61	8.45	-0.61
66	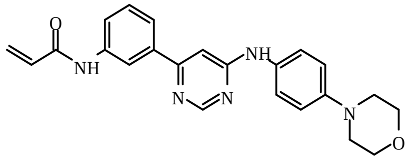	4	training	6.74	9.14	-0.01	9.14	-0.01
67	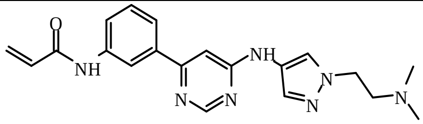	4	training	6.51	8.85	-0.30	8.85	-0.30
68	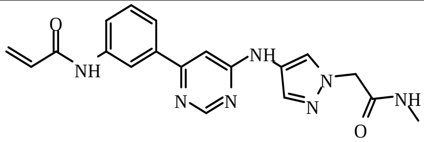	4	training	6.86	8.73	-0.42	8.73	-0.42
69	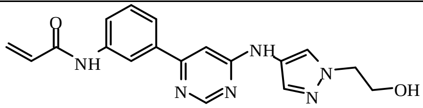	4	test	6.22	6.75	-2.40	6.75	-2.40
70	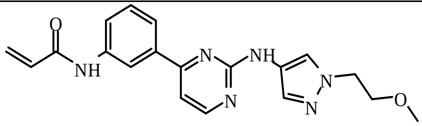	4	training	7.26	9.08	-0.14	9.08	-0.14
71	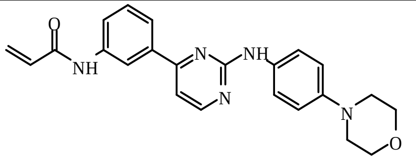	4	training	7.52	9.07	-0.15	9.07	-0.15
72	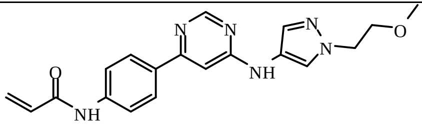	4	training	7.17	8.79	-0.43	8.79	-0.43
73	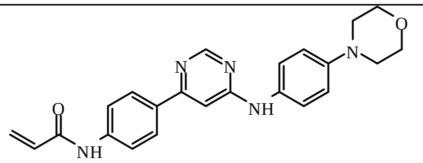	4	training	6.95	9.26	0.04	9.26	0.04

74	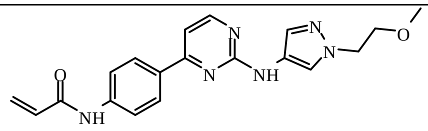	4	training	8.04	9.11	-0.13	9.11	-0.13
75	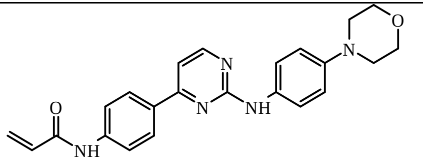	4	training	8.77	9.18	-0.12	9.18	-0.12

pIC_{50}(A): Predicted Activity (Field-based).

pIC_{50}(B): Predicted Activity (Atom-based).
